# Dual Targeting of EZH2 and LSD1 Suppresses Hepatocellular Carcinoma via Disruption of Sonic Hedgehog Signaling

**DOI:** 10.3390/ijms27093886

**Published:** 2026-04-27

**Authors:** HongDuck Yun, Ponmari Guruvaiya, Olena Levurdiak, Alexei G. Basnakian, Marjan Boerma, Stephen Safe, KyoungHyun Kim

**Affiliations:** 1 Department of Pharmacology and Toxicology, College of Medicine, University of Arkansas for Medical Sciences, Little Rock, AR 72225, USA; 2 Department of Pharmaceutical Sciences, College of Pharmacy, University of Arkansas for Medical Sciences, Little Rock, AR 72225, USA; 3 Department of Veterinary Physiology and Pharmacology, Texas A&M University, College Station, TX 77843, USA

**Keywords:** hepatocellular carcinoma, dual inhibition, EZH2, LSD1, SHH, STAT3, GLI1

## Abstract

Hepatocellular carcinoma (HCC) is a highly aggressive malignancy with poor prognosis and limited therapeutic options. Although epigenetic dysregulation is a hallmark of HCC, rational combinatorial targeting strategies remain incompletely defined. Here, we identify cooperative oncogenic functions of the chromatin modifiers enhancer of zeste homolog 2 (*EZH2*) and lysine-specific demethylase 1 (*LSD1*) in HCC. Analysis of the TCGA-LIHC cohort revealed that co-elevated *EZH2* and *LSD1* expressions are significantly associated with reduced overall survival. Gene set enrichment analysis demonstrated enrichment of Sonic Hedgehog (SHH) signaling and stress-responsive transcriptional programs in tumors with high *EZH2/LSD1* expression. Functionally, dual pharmacological inhibition of *EZH2* (GSK126) and *LSD1* (SP2509) suppressed HCC cell proliferation, induced G1-phase arrest, and enhanced apoptosis, as evidenced by increased caspase-3/7 activity and decreased pro-caspase levels. Dual inhibition also impaired migration, invasion, tumor sphere formation, and stemness-associated gene expression. Mechanistically, co-targeting disrupted SHH signaling through the suppression of *GLI1* expression. Chromatin immunoprecipitation revealed reduced *EZH2*, *LSD1*, and *STAT3* occupancy at the *GLI1* promoter following dual inhibition, leading to the repression of *GLI1* and its downstream targets. Collectively, these findings demonstrate that *EZH2* and *LSD1* cooperatively sustain *GLI1*-dependent SHH signaling in HCC, and that dual epigenetic inhibition represents a mechanistically defined therapeutic strategy.

## 1. Introduction

Hepatocellular carcinoma (HCC) is the most prevalent form of primary liver cancer and ranks as the third leading cause of cancer-related mortality worldwide [1,2]. Despite recent therapeutic advancements, the overall prognosis for HCC remains poor, largely due to late-stage diagnosis, high recurrence rates, and the emergence of resistance to systemic therapies [3,4]. First-line treatment options for unresectable HCC include multi-kinase inhibitors such as sorafenib and lenvatinib, as well as combination immunotherapies, notably the immune checkpoint inhibitor atezolizumab in conjunction with the VEGF-A inhibitor bevacizumab [5,6]. These regimens have led to meaningful improvements in overall survival. However, their clinical efficacy remains limited, with objective response rates of approximately 27%, and most patients ultimately develop resistance within 12 to 18 months. These limitations highlight an urgent need for novel therapeutic strategies that more effectively target the molecular pathways driving HCC progression and therapeutic resistance.

Recent studies have underscored the pivotal role of epigenetic dysregulation, including histone modifications, DNA methylation, and chromatin remodeling, in the progression of HCC, as well as in its intratumoral heterogeneity and resistance to therapy [7,8]. Among the key epigenetic regulators, *EZH2*, the catalytic subunit of the Polycomb Repressive Complex 2 (PRC2) responsible for tri-methylation of histone H3 at lysine 27 [9], plays a central role in silencing tumor suppressor genes. *EZH2* is frequently overexpressed in HCC and has been strongly associated with aggressive disease phenotypes and poor clinical outcomes [10,11,12]. Likewise, lysine-specific demethylase 1 (*LSD1*/KDM1A) [13], a flavin adenine dinucleotide (FAD)-dependent histone demethylase that removes mono- and di-methyl groups from H3K4 and H3K9, is commonly upregulated in HCC. *LSD1* promotes tumor progression by maintaining cancer stemness, reprogramming cellular metabolism, including glycolysis and mitochondrial function, and is similarly linked to unfavorable prognosis [14,15,16,17].

*EZH2* and *LSD1* have each been independently validated as oncogenic drivers and promising therapeutic targets in HCC; however, their dual inhibition remains underexplored. Given their complementary roles in epigenetic regulation and their promotion of malignant phenotypes, including enhanced proliferation, migration, and maintenance of cancer stem cell features, we investigated whether dual inhibition of EZH2 and LSD1 could enhance antitumor efficacy in HCC. To this end, we treated HCC cell models with GSK126, a selective EZH2 inhibitor [18,19], and SP2509, a selective LSD1 inhibitor [20,21,22]. Dual inhibition significantly suppressed cell proliferation, induced cell cycle arrest and apoptosis, and reduced migration, invasion, and tumor sphere formation. Mechanistically, dual inhibition disrupted the *STAT3*–*GLI1* regulatory axis, linking *STAT3* signaling to Sonic Hedgehog (SHH) pathway activation. Specifically, co-targeting *EZH2* and *LSD1* reduced *STAT3* occupancy at the *GLI1* promoter, leading to suppression of *GLI1* transcription and attenuation of downstream SHH signaling. Collectively, these findings demonstrate that coordinated epigenetic regulation by *EZH2* and *LSD1* sustains *STAT3*-driven SHH signaling in HCC, and that dual inhibition effectively disrupts this oncogenic circuitry, highlighting a mechanistically defined therapeutic strategy with translational potential. 

## 2. Results

### 2.1. Prognostic Significance of EZH2 and LSD1 Expression in HCC: Evidence from TCGA-LIHC Survival Analysis

**Figure 1:**
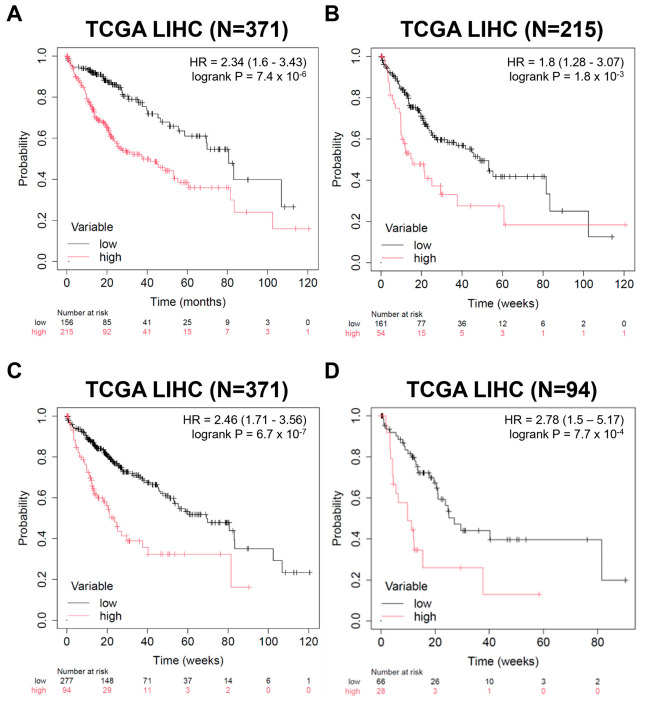
High *EZH2* and *LSD1* expressions are strongly correlated with poor prognosis in HCC patients. Kaplan–Meier survival plots were generated using the TCGA LIHC cohort. (**A**) Overall survival of HCC patients was dichotomized by *EZH2* expression level (*EZH2* High, *EZH2*^H^, N = 215; *EZH2* Low, *EZH2*^L^, N = 156). (**B**) Overall survival of HCC patients expressing high *EZH2* levels (N = 215) was further stratified by *LSD1* expression level (*EZH2*^H^*LSD1*^H^, N = 54; EZH2^H^LSD1^L^, N = 161). (**C**) The overall survival of HCC patients was dichotomized by *LSD1* expression level (*LSD1* High, *LSD1*^H^, N = 94; *LSD1* Low, *LSD1*^L^, N = 277). (**D**) Overall survival of HCC patients expressing high *LSD1* levels (N = 94) was further stratified by *LSD1* expression level (*LSD1*^H^*EZH2*^H^, N = 28; *LSD1*^H^*EZH2*^L^, N = 66). The log-rank test was used to assess statistical significance. Hazard ratios (HR) and *p*-values are indicated on the plots.

To assess the clinical relevance of *EZH2* and *LSD1* in HCC, we performed Kaplan–Meier analysis of the TCGA-LIHC cohort using an outcome-based optimal cut-off approach. This data-driven method better discriminates survival outcomes than an arbitrary median split and identifies clinically meaningful high-risk groups [23]. Patients with high *EZH2* expression exhibited significantly worse overall survival compared with those with low *EZH2* expression (log rank *p* = 7.4 × 10^−6^; HR = 2.34) (Figure 1A). Similarly, high *LSD1* expression was associated with reduced survival relative to low *LSD1* expression (log rank *p* = 6.7 × 10^−7^; HR = 2.46) (Figure 1C). To determine whether co-expression of these enzymes further refined prognosis, we performed hierarchical stratification analyses. Among patients with high *EZH2* expression (N = 215), those with concomitantly high *LSD1* levels (N = 54) had significantly poorer survival than those with low *LSD1* expression (log rank *p* = 0.0018; HR = 1.98) (Figure 1B). Likewise, within the group of patients with high *LSD1* expression (N = 94), individuals with high *EZH2* levels (N = 28) had worse overall survival than those with low *EZH2* expression (N = 66) (log rank *p* = 0.00077; HR = 2.78) (Figure 1D). Together, these results demonstrate that co-elevation of *EZH2* and *LSD1* identifies a subgroup of HCC patients with the most unfavorable prognosis, supporting their cooperative role in aggressive disease. These findings provide a strong rationale for dual targeting of *EZH2* and *LSD1* as a therapeutic strategy in HCC. Although several selective inhibitors of *EZH2* or *LSD1* are currently available and undergoing clinical evaluation in other malignancies [24,25], the therapeutic potential of their dual inhibition has not yet been systematically investigated in HCC.

### 2.2. Dual Inhibition of EZH2 and LSD1 Enhances Suppression of HCC Proliferation and Perturbs Cell Cycle Control

**Figure 2:**
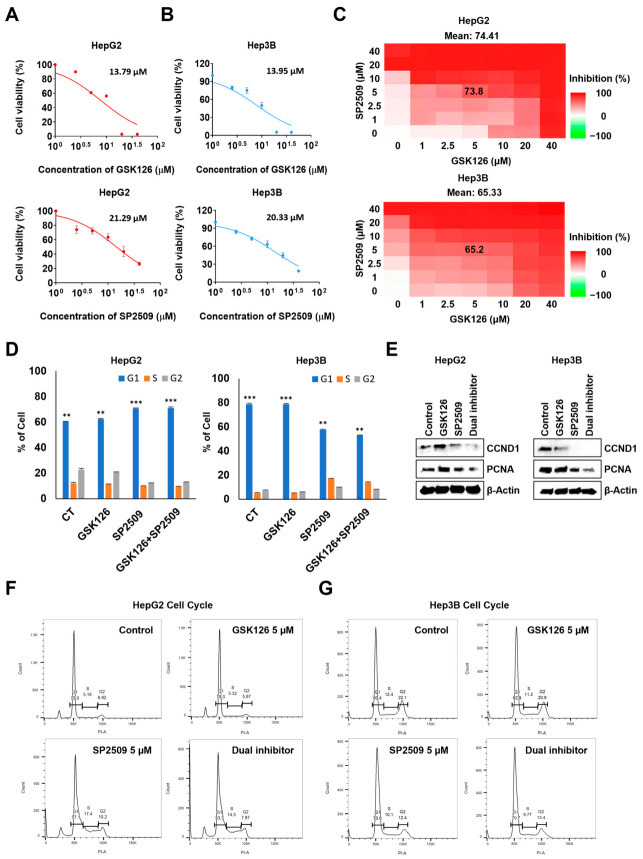
Dual inhibition of *EZH2* and *LSD1* suppresses HCC cell proliferation and alters cell cycle progression. (**A**,**B**) Dose–response curves for the *EZH2* inhibitor GSK126 and the *LSD1* inhibitor SP2509, with calculated IC_50_ values in HepG2 and Hep3B cells after 48 h of treatment. (**C**) Two-dimensional dose–response matrix depicting the dual inhibition effects of GSK126 and SP2509 on cell viability. Red regions represent increasing levels of growth inhibition. (**D**) Representative cell-cycle profiles analyzed by propidium iodide (PI) staining and flow cytometry following 48 h treatment with 5 µM GSK126 and 5 µM SP2509. (**E**) Immunoblot analysis showing reduced expression of proliferation-associated proteins CCND1 and PCNA following single-agent or dual treatment; β-Actin was used as a loading control. (**F**,**G**) Quantification of cell-cycle distribution demonstrating treatment-induced alterations in G1-, S-, and G2-phase populations in HepG2 and Hep3B cells after dual GSK126 and SP2509 treatment. All quantitative data are presented as mean ± SD (** *p* < 0.01, *** *p* < 0.001 by two-way ANOVA).

To evaluate the antiproliferative effects of *EZH2* and *LSD1* inhibition, HepG2 and Hep3B cells were treated for 48 h with increasing concentrations of the *EZH2* inhibitor GSK126 or the *LSD1* inhibitor SP2509. MTT assays demonstrated dose-dependent growth inhibition in both cell lines. The calculated IC_50_ values were 13.79 µM (GSK126) and 21.29 µM (SP2509) in HepG2 cells, and 13.95 µM (GSK126) and 20.33 µM (SP2509) in Hep3B cells (Figure 2A,B).

To determine whether dual inhibition produced additive or synergistic effects, we generated two-dimensional dose–response matrices (0–40 µM for each drug) in both cell lines and analyzed drug interactions using SynergyFinder [26]. Heatmap visualization revealed concentration-dependent inhibition of viability with the drug combination in both HepG2 and Hep3B cells (Figure 2C). Synergy analysis demonstrated positive interactions across multiple models in HepG2 cells (Loewe = 8.98, HSA = 11.17, Bliss = 1.77, ZIP = 1.07), accompanied by a high Combination Sensitivity Score (CSS = 92.09) and substantial contributions from both agents (RI_1_ = 65.27 for GSK126; RI_2_ = 40.16 for SP2509) (Supplementary Tables S3 and S4). Similarly, Hep3B cells exhibited robust synergy across all models (ZIP = 9.55, Loewe = 8.66, HSA = 16.34, Bliss = 8.17) with strong dual inhibition sensitivity (CSS = 84.35) and comparable contributions from GSK126 (RI_1_ = 37.80) and SP2509 (RI_2_ = 42.92) (Supplementary Tables S5 and S6). Collectively, these results indicate that dual inhibition of *EZH2* and *LSD1* suppresses HCC cell viability more effectively than either agent alone, exhibiting additive to modestly synergistic effects depending on the analytical model.

Based on these findings, we selected 5 µM GSK126 + 5 µM SP2509 as the working combination for subsequent mechanistic studies. The dose-dependent inhibitory effects on *EZH2* and *LSD1* enzymatic activities were validated by immunoblot analysis of their respective histone substrates, demonstrating reduced H3K27me3 and altered H3K4me2 levels following dual treatment (Supplementary Figure S1A,B). This concentration fell within the high-effect region of the 2D matrix while remaining experimentally practical. At this dose, the combination produced strong and reproducible antiproliferative activity (mean inhibition: ~74% in HepG2 and ~65% in Hep3B), providing sufficient dynamic range to assess downstream cell-cycle effects without reaching near-maximal toxicity levels.

Cell-cycle analysis by propidium iodide (PI) staining and flow cytometry revealed cell-line-specific responses to dual treatment. In HepG2 cells, the dual inhibition significantly decreased the G1 population with a corresponding increase in S-phase cells (Figure 2D, left; Figure 2F), whereas in Hep3B cells, treatment led to accumulation in both G1 and G2 phases (Figure 2D, right; Figure 2G), suggesting checkpoint arrest at distinct stages depending on cellular context. Consistent with these findings, dual inhibition more effectively reduced expression of cell proliferation-related proteins, including PCNA and Cyclin D1, compared with single treatment (Figure 2E; Supplementary Figure S2A,B), indicating enhanced suppression of cell-cycle progression.

Together, these results demonstrate that dual inhibition of *EZH2* and *LSD1* significantly suppresses HCC cell proliferation by perturbing cell-cycle regulation and downregulating key proliferation-associated proteins.

### 2.3. Dual Targeting of EZH2 and LSD1 Effectively Restrains HCC Cell Migration and Invasion

**Figure 3:**
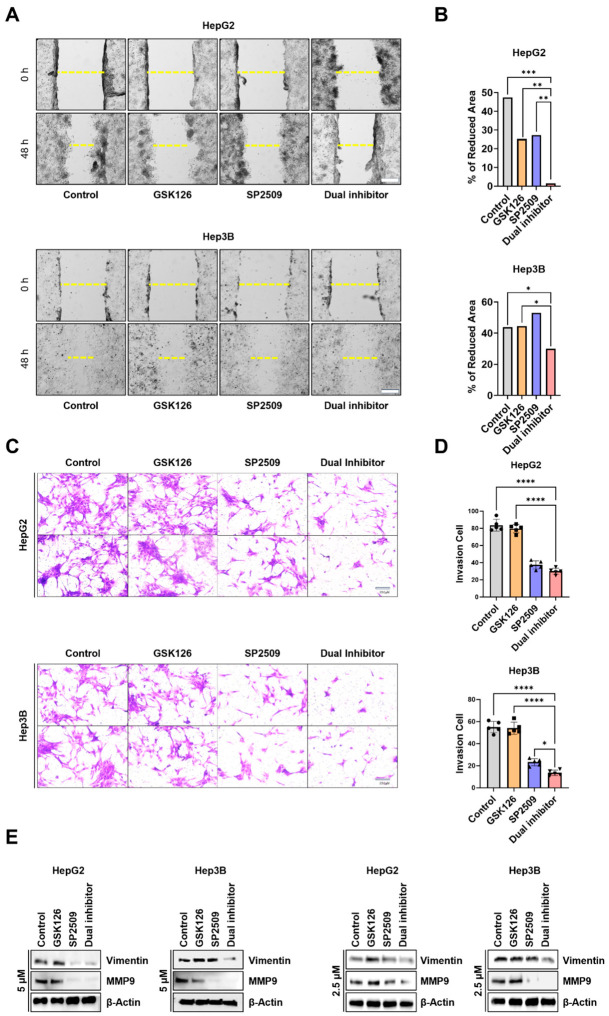
Effects of Dual treatment on HCC cell migration and invasion. (**A**,**B**) Representative images of scratch wound healing assays and % of reduced area in HepG2 and Hep3B cells treated with GSK126 5 µM, SP2509 5 µM, or Dual inhibition for 48 h. Wound width was measured at 0 and 48 h and expressed as percent closure (×20, Scale bar, 100 µm). (**C**,**D**) HepG2 and Hep3B cells were treated with GSK126 5 µM, SP2509 5 µM, or Dual treatment for 48 h, followed by invasion assays using Matrigel-coated Trans-well membrane for 24 h from the four group. (magnification, ×200, scale bar, 250 µm). (**E**) Immunoblotting analysis of EMT-related migration markers vimentin and MMP9 in HepG2 and Hep3B cells after with GSK126 2.5, 5 µM, SP2509 2.5, 5 µM, or Dual treatment for 48 h. All Data represent mean ± SD from three independent experiments. All quantification results are shown as the mean ± SD (* *p* < 0.05, ** *p* < 0.01, *** *p* < 0.001, **** *p* < 0.0001 by two-way ANOVA).

To determine the functional impact of dual *EZH2* and *LSD1* inhibition on HCC cell behavior, we evaluated cell migration, invasion, and the expression of migration/invasion-associated proteins following treatment with GSK126 and SP2509. Scratch wound-healing assays revealed that combined treatment markedly impaired the migratory capacity of both HepG2 and Hep3B cells compared with single-agent or control conditions. At 48 h, cells treated with 5 µM GSK126 and 5 µM SP2509 exhibited significantly reduced wound closure, indicating strong inhibition of lateral cell movement (Figure 3A). Quantitative analysis confirmed a significant decrease in migration area in the dual treatment group relative to either monotherapy (Figure 3B). To distinguish anti-migratory effects from cytotoxicity, assays were repeated at lower, minimally cytotoxic concentrations (1 µM GSK126 plus 1 µM SP2509 and 2.5 µM GSK126 plus 2.5 µM SP2509, respectively). Under these conditions, dual inhibition consistently suppressed cell migration without significantly affecting cell viability (Supplementary Figures S3 and S4), supporting a direct effect on migratory capacity.

Consistent with these findings, transwell Matrigel invasion assays demonstrated that dual treatment significantly reduced the invasive potential of both HCC cell lines at 24 h. Notably, fewer cells traversed the Matrigel-coated membrane following dual treatment compared with single-agent or vehicle controls (Figure 3C,D), indicating enhanced suppression of extracellular matrix invasion. Immunoblot analysis further showed that dual inhibition led to a more pronounced reduction in key regulators of migration and invasion, including vimentin and MMP9, compared with either inhibitor alone (Figure 3E; Supplementary Figure S5C–F). At lower concentrations, invasion was effectively suppressed in Hep3B cells, whereas HepG2 cells exhibited a comparatively reduced response (Supplementary Figure S5A,B), suggesting potential cell line-specific sensitivity.

Collectively, these results demonstrate that dual *EZH2* and *LSD1* inhibition not only suppresses HCC cell proliferation but also robustly impairs migration and invasion, supporting a broader anti-tumor effect of this combinatorial epigenetic strategy.

### 2.4. Dual Inhibition of EZH2 and LSD1 Enhances Caspase-Dependent Apoptosis in HCC Cells

**Figure 4:**
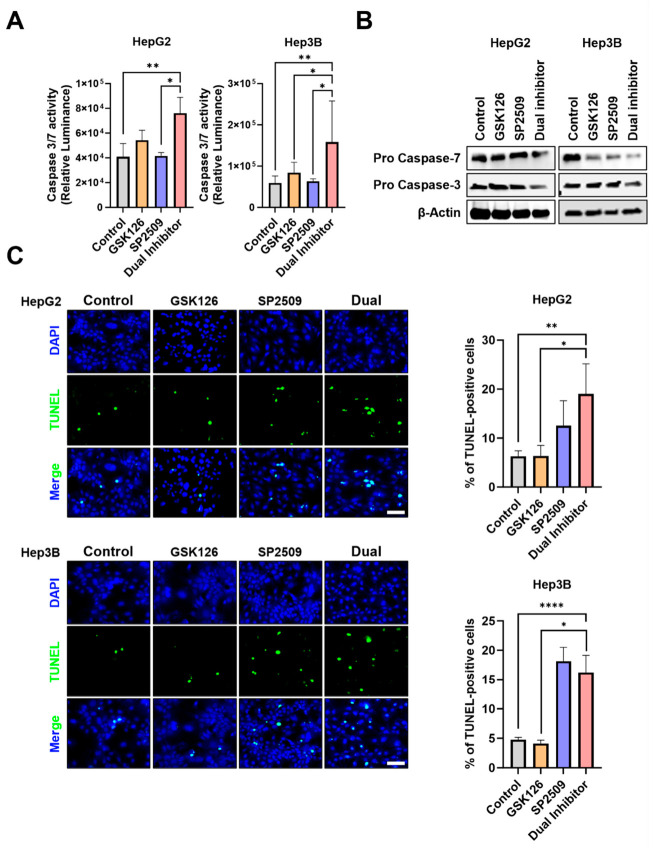
Dual inhibition of *EZH2* and *LSD1* enhances apoptosis in HCC cells. (**A**) Caspase 3/7 activity assay in HepG2 and Hep3B cells after 24 h treatment with GSK126 5 µM, SP2509 5 µM, or Dual treatment. Relative luminescence is shown to indicate caspase activity. (**B**) Immunoblotting analysis of pro-caspase 3 and 7 expression under the same treatments. Dual treatment led to a significant increase in caspase activity and a decrease in pro-caspase levels, indicating activation of the caspase cascade. (**C**) HepG2 and Hep3B cells were treated with 5 µM GSK126, 5 µM SP2509, or the dual treatment for 48 h, followed by TUNEL staining to detect apoptotic DNA fragmentation (green). Nuclei were counterstained with DAPI (blue). Representative fluorescence images are shown for each condition. Cells treated with both GSK126 and SP2509 exhibited a marked increase in TUNEL-positive nuclei, indicating elevated apoptotic DNA damage following dual inhibition. Images were captured at ×200 magnification, and scale bars represent 50 µm. All quantification results are presented as mean ± SD (* *p* < 0.05, ** *p* < 0.01, **** *p* < 0.0001 by one-way ANOVA).

Pharmacological inhibition of either *EZH2* or *LSD1* has been reported to suppress HCC growth, in part by inducing apoptosis [27,28]. To determine whether dual inhibition further enhances this effect, we evaluated apoptotic responses in HepG2 and Hep3B cells following treatment with GSK126 and SP2509. After 24 h of dual treatment, caspase-3/7 activity was significantly elevated in both cell line dual treatments with control or single treatments (Figure 4A), indicating activation of the executioner caspase pathway. Consistent with this, immunoblot analysis revealed a marked reduction in pro-caspase-3 and pro-caspase-7 levels in Dual-treated cells, whereas little or no reduction was observed in control or single treated groups (Figure 4B; Supplementary Figure S6A,B), further supporting enhanced caspase activation by the Dual inhibitor.

To corroborate these findings, TUNEL staining was performed after 48 h of treatment to assess DNA fragmentation. Whereas control cells exhibited minimal TUNEL positivity, dual-treated HepG2 and Hep3B cells displayed a substantial increase in green fluorescent TUNEL-positive nuclei, consistent with apoptotic DNA cleavage. Quantitative analysis confirmed a significant rise in TUNEL-positive cells following dual inhibition of *EZH2* and *LSD1* in both cell lines (Figure 4C).

Collectively, these results demonstrate that dual inhibition of *EZH2* and *LSD1* promotes apoptotic cell death in HCC cells through activation of a caspase-dependent pathway.

### 2.5. Dual Inhibition of EZH2 and LSD1 Suppresses Tumor Sphere Formation and Attenuates Stemness-Associated Signatures in HCC Cells

**Figure 5:**
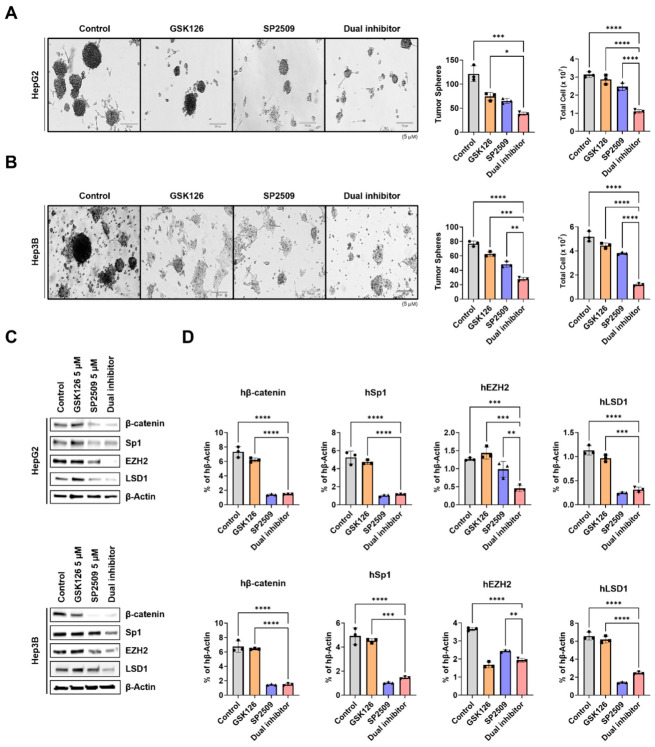
Dual inhibition reduces tumor sphere formation and cancer stem cell marker expression. (**A**,**B**) Representative images and quantification of tumor spheres formed by HepG2 and Hep3B cells cultured with GSK126 5 µM, SP2509 5 µM, or dual inhibitor treatment. Cells were seeded in 96-well plates at a density of 5 × 10^2^ cells per well. Sphere numbers per well were counted after 14 days (magnification, ×200, scale bar, 100 µm). (**C**) Immunoblotting analysis showing the expression of cancer stem cell markers β-catenin, Sp1, *EZH2*, and *LSD1* in HepG2 and Hep3B following the same treatments. (**D**) The corresponding mRNA levels were determined by qRT-PCR. mRNA levels were normalized to hβ-actin as control. All quantification results are shown as the mean ± SD (* *p* <0.05, ** *p* < 0.01, *** *p* < 0.001, **** *p* < 0.0001 by Ordinary one-way ANOVA and Two-way ANOVA).

*EZH2* and *LSD1* are well-established regulators of cancer stemness and have been implicated in the maintenance of self-renewal across multiple malignancies, including HCC [29,30,31]. To determine whether concurrent inhibition of these epigenetic enzymes disrupts cancer stem-like properties, we performed a tumor sphere formation assay using HepG2 and Hep3B cells. Compared with vehicle control or either single-agent treatment, dual *EZH2* and *LSD1* inhibition significantly reduced both the number of tumor spheres formed and the number of cells within each sphere in HepG2 (Figure 5A) and Hep3B (Figure 5B) cultures (representative images shown). To exclude potential confounding effects of cytotoxicity, additional experiments were conducted at lower inhibitor concentrations. Under these conditions, dual inhibition consistently suppressed tumor sphere formation and reduced cell numbers per sphere (Supplementary Figure S8D,E), confirming a direct effect on cancer stem-like properties.

Consistent with this phenotypic suppression of stemness, immunoblot analysis demonstrated that dual treatment markedly decreased the protein levels of key stemness-associated regulators, including β-catenin, Sp1, *EZH2*, and *LSD1* (Figure 5C; Supplementary Figure S7A,B). Parallel reductions in the corresponding mRNA transcripts were also observed (Figure 5D), indicating coordinated transcriptional repression of stemness programs. Additionally, we detected decreased mRNA expression of other core stem cell markers, including Oct4 and NANOG in HepG2 cells following dual treatment (Supplementary Figure S7C).

To assess whether dual inhibition could also disrupt pre-established tumor spheres, HepG2 and Hep3B spheres were first cultured for 14 days to allow full maturation, after which they were treated with either single agents or the dual treatment. Notably, dual treatment produced a significantly greater reduction in cell viability within these pre-formed spheres compared with dual, single or control, demonstrating a potent ability to destabilize established tumor spheroids in vitro (Supplementary Figure S8A–C). Taken together, these findings demonstrate that simultaneous inhibition of *EZH2* and *LSD1* impairs both the self-renewal capacity and the cancer stem-like phenotype of HCC cells. These results underscore the therapeutic potential of dual epigenetic targeting as a strategy to suppress cancer stemness, improve treatment durability, and mitigate the risk of tumor recurrence.

### 2.6. Dual Inhibition of EZH2 and LSD1 Suppresses Sonic Hedgehog Signaling via Attenuation of the STAT3–GLI1 Axis in HCC

To identify key signaling pathways commonly regulated by *EZH2* and *LSD1* in hepatocellular carcinoma (HCC), Gene Set Enrichment Analysis (GSEA) was performed using the TCGA-LIHC dataset. HCC samples were stratified into *EZH2*^High^ vs. *EZH2*^Low^ and *LSD1*^High^ vs. *LSD1*^Low^ groups, as shown in Figure 1A,B. GSEA was conducted using the Hallmark gene sets to systematically compare pathway enrichment between these groups. Notably, the UV_RESPONSE_UP gene signature was prominently enriched in both *EZH2*^High^ and *LSD1*^High^ groups (Supplementary Figure S9A,B), suggesting that elevated expression of these epigenetic modifiers is associated with activation of stress-responsive transcriptional programs. This gene set includes genes involved in cell cycle regulation, DNA damage response, and apoptosis. These observations align with our experimental findings demonstrating altered cell cycle progression and increased apoptosis following dual pharmacological inhibition of *EZH2* and *LSD1* (Figure 2 and Figure 4). Together, these data support the notion that *EZH2* and *LSD1* cooperatively regulate stress-adaptive transcriptional networks that may promote tumor cell survival and therapeutic resistance in HCC.

**Figure 6:**
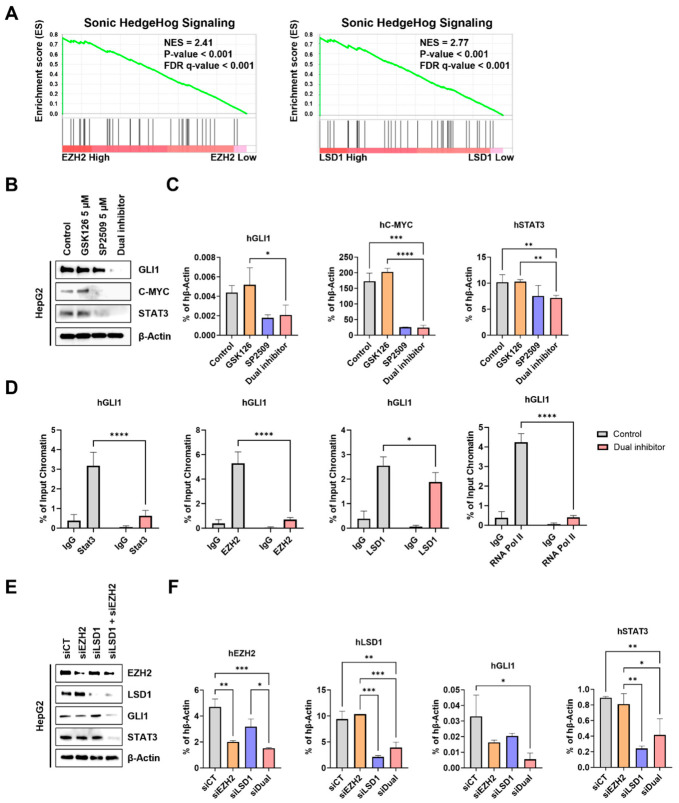
Dual inhibition of *EZH2* and *LSD1* suppresses Sonic Hedgehog signaling through disruption of the *STAT3*–*GLI1* signaling axis. (**A**) Gene Set Enrichment Analysis (GSEA) plots showing the enrichment of Sonic Hedgehog Signaling gene sets in *EZH2*^High/Low^ and *LSD1* ^High/Low^ groups. The GSEA analysis was performed with the Hallmark gene signature. (**B**,**C**) Immunoblotting and qPCR analyses of key SHH pathway components, including *GLI1*, C-MYC, and *STAT3*, an upstream regulator of *GLI1* following dual pharmacological targeting of *EZH2* and *LSD1* (5 µM GSK126 and 5 µM SP2509 for 48 h). (**D**) ChIP-PCR analysis showing *STAT3* and RNA polymerase II occupancy at the *GLI1* promoter region (−1021 to −752 bp from the transcription start site). Enrichment is presented as a percentage of input chromatin. Dual inhibition was performed with 5 µM GSK126 and 5 µM SP2509 for 16 h. (**E**,**F**) Immunoblotting and qPCR analysis of *EZH2*, *LSD1*, *GLI1* and *STAT3* in HepG2 cells transfected with siRNAs targeting *EZH2* (siEZH2) or *LSD1* (siLSD1), compared with scrambled control (siCT), validating genetic modulation of the pathway. All quantification results are shown as the mean ± SD (* *p* < 0.05, ** *p* < 0.01, *** *p* < 0.001, **** *p* < 0.0001 by two-way ANOVA).

Among commonly enriched pathways, SHH signaling pathway emerged as a prominent target co-regulated by *EZH2* and *LSD1* (Figure 6A). To determine whether pharmacological inhibition of these enzymes affects SHH pathway activity, we examined the expression of key pathway components. Dual inhibition of *EZH2* and *LSD1* markedly reduced GLI1, C-MYC and STAT3 at both the protein and mRNA levels (Figure 6B,C; Supplementary Figure S9C) [32,33,34]. Notably, STAT3, a transcription factor previously implicated in upstream regulation of *GLI1* in multiple cancer types, was also significantly decreased, although its role in HCC remains incompletely defined [35,36]. In silico analysis of the *GLI1* promoter identified five putative STAT3-binding sites within 1 kb upstream of the transcription start site [37]. To investigate whether EZH2, LSD1, and STAT3 cooperatively regulate GLI1 transcription, we performed chromatin immunoprecipitation (ChIP) assays using antibodies against EZH2, LSD1, STAT3, and RNA polymerase II. Robust occupancy of all four factors was detected at the *GLI1* promoter region (Figure 6D), supporting the formation of an active transcriptional complex. Consistent with these findings, ENCODE datasets demonstrated co-localization of *EZH2* and *LSD1* at the *GLI1* promoter in HepG2 cells (Supplementary Figure S10). These observations are further supported by prior studies demonstrating that *EZH2* and *LSD1* can function as transcriptional co-activators and interact with *STAT3* [38,39,40,41,42,43]. Importantly, dual inhibition significantly reduced the occupancy of *EZH2*, *LSD1*, *STAT3*, and RNA polymerase II at the *GLI1* promoter within 16 h, indicating that these epigenetic regulators are required for transcriptional activation of *GLI1*.

Functional validation was further performed using STATTIC, a selective *STAT3* inhibitor [44]. STATTIC treatment significantly decreased *GLI1* expression and reduced levels of Cyclin D1, a well-established *GLI1* downstream target (Supplementary Figure S11), confirming that *STAT3* positively regulates *GLI1* signaling in HepG2. Furthermore, siRNA-mediated knockdown experiments demonstrated that simultaneous depletion of *EZH2* and *LSD1* significantly reduced *GLI1* and *STAT3* expressions, whereas single knockdown produced only partial effects (Figure 6E,F). Conversely, ectopic overexpression of *EZH2* and *LSD1* increased *STAT3*, *GLI1*, C-MYC, and Cyclin D1 expression at both mRNA and protein levels (Supplementary Figure S12A–C), supporting a cooperative role of these epigenetic modifiers in sustaining *STAT3*–*GLI1* signaling.

**Figure 7:**
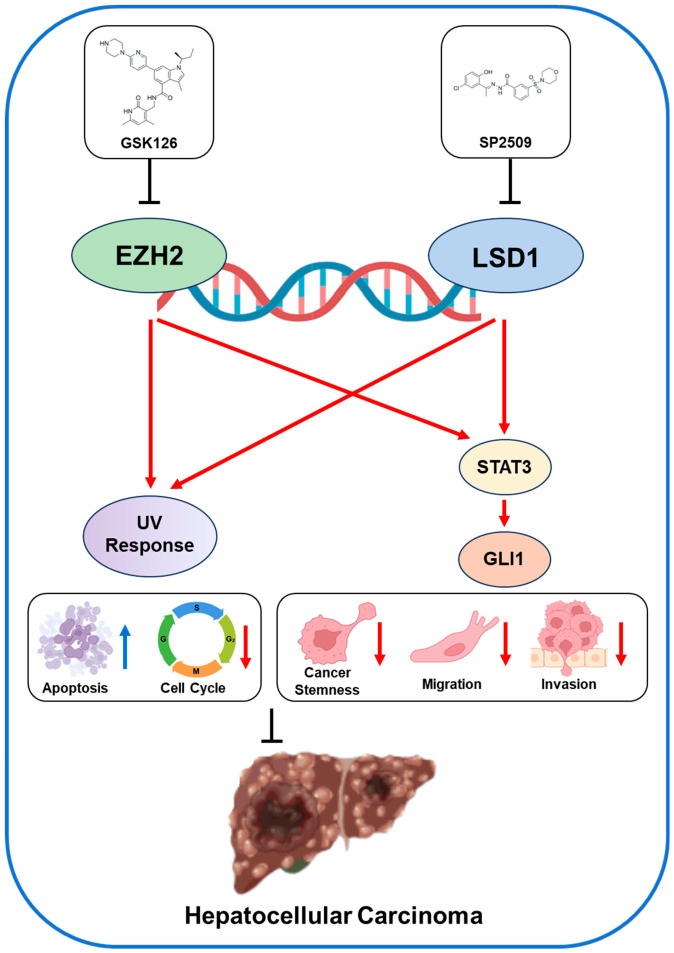
Schematic model illustrating cooperative regulation of UV response and Sonic Hedgehog signaling by *EZH2* and *LSD1* in HCC.

Collectively, these findings demonstrate that *EZH2* and *LSD1* coordinately regulate the SHH pathway in HCC. Both pharmacological inhibition and genetic depletion suppress SHH signaling by disrupting the *STAT3*-*GLI1* signaling axis, thereby revealing a previously unrecognized epigenetic control point in Hedgehog-driven oncogenic signaling (Figure 7).

The diagram depicts the proposed mechanism by which the epigenetic modifiers *EZH2* and *LSD1* coordinately regulate transcriptional programs that promote HCC progression. Both enzymes associate with chromatin and modulate gene expression, influencing two major pathways: (i) stress-responsive transcriptional programs, including the UV response pathway, and (ii) Sonic Hedgehog (SHH) signaling. Dual inhibition of *EZH2* and *LSD1* suppresses UV response gene expression and disrupts SHH pathway activity. Mechanistically, SHH signaling is attenuated through inhibition of the *EZH2*/*LSD1*/*STAT3*–*GLI1* signaling axis. Downregulation of these pathways may induce impaired cell cycle progression, reduced proliferation, decreased migration and invasion, and enhanced apoptosis, ultimately suppressing liver tumor growth. Upward and downward arrows indicate increased or decreased biological activity, respectively.

## 3. Discussion

HCC remains a highly lethal malignancy characterized by limited therapeutic responsiveness and frequent resistance to systemic therapies. Epigenetic dysregulation is increasingly recognized as a central driver of HCC progression; however, rational combinatorial targeting strategies remain insufficiently defined. In this study, we identify a cooperative epigenetic mechanism by which *EZH2* and *LSD1* sustain oncogenic transcriptional programs and demonstrate that their dual inhibition suppresses malignant phenotypes in HCC cells. Mechanistically, our data support a model in which *EZH2* and *LSD1* converge on a *STAT3*–*GLI1* transcriptional axis, thereby linking chromatin regulation to GLI1-dependent gene expression programs.

A key finding of this study is that co-elevated *EZH2* and *LSD1* expressions identify a subset of HCC patients with particularly poor clinical outcomes. While both enzymes have been independently implicated in tumor progression, our hierarchical stratification analysis indicates that their combined expression provides additional prognostic resolution beyond either factor alone. These observations are consistent with emerging evidence that epigenetic regulators often function in coordinated networks rather than as isolated drivers, and they provide a clinical rationale for combinatorial targeting approaches.

Functionally, dual inhibition of *EZH2* and *LSD1* produced greater suppression of proliferation, migration, invasion, and tumor sphere formation compared with single-agent treatment. These effects were accompanied by cell cycle perturbation and induction of apoptosis, consistent with prior studies targeting either enzyme individually. Importantly, our data indicates that the interaction between *EZH2* and *LSD1* is additive-to-enhanced, rather than strongly synergistic, based on matrix-based drug interaction analyses. This distinction is critical, as it suggests that the therapeutic benefit of dual targeting arises from complementary disruption of distinct but convergent chromatin-regulatory functions rather than classical pharmacologic synergy.

Mechanistically, we identify *GLI1* as a central transcriptional output regulated by *EZH2* and *LSD1*. While SHH signaling has been implicated in HCC progression and stemness, direct evidence linking epigenetic modifiers to *GLI1* regulation in this context has been limited. Our findings demonstrate that *EZH2*, *LSD1*, and *STAT3* co-occupied the *GLI1* promoter, and that dual inhibition reduces the recruitment of these factors along with RNA polymerase II, leading to transcriptional repression of *GLI1*. In addition, pharmacological inhibition of *STAT3* recapitulated *GLI1* suppression, and genetic depletion of *EZH2* and *LSD1* produced concordant effects. Together, these results support a model in which *EZH2* and *LSD1* facilitate *STAT3*-dependent transcription of *GLI1*.

Importantly, our data refine the interpretation of SHH pathway involvement. Rather than indicating global inhibition of canonical SHH signaling, our results support suppression of a *GLI1*-centered transcriptional axis, suggesting that *EZH2* and *LSD1*, together with *STAT3*, contribute to *GLI1* activation in HCC. Beyond transcriptional regulation, dual inhibition also reduced phenotypes associated with tumor aggressiveness, including migration, invasion, and cancer stem-like properties. However, these findings should be interpreted with caution, as cytotoxic effects at higher concentrations or prolonged exposure may partially contribute to these outcomes. To address this, we performed additional experiments at lower, minimally cytotoxic doses, which continued to inhibit migration and invasion, supporting a direct effect on motility pathways.

Several additional limitations should be considered. First, our findings are based on established HCC cell lines and therefore do not capture the full complexity of tumor heterogeneity or microenvironmental interactions. Second, we did not include non-transformed hepatocyte models, limiting our ability to assess therapeutic selectivity and potential toxicity. While prior studies of *EZH2* and *LSD1* inhibitors demonstrate context-dependent antitumor activity in cancer models [20,21,22], our data do not directly address selectivity in HCC. Accordingly, we have moderated our claims regarding selective anticancer effects and explicitly acknowledge this limitation. Third, although genetic knockdown experiments support target specificity, future studies using additional pharmacologic tools or orthogonal approaches will be important to confirm these findings. In addition, while multivariate survival analyses were not performed in this study, prior independent HCC cohorts have demonstrated that *EZH2* and *LSD1* are independent prognostic factors, supporting the clinical relevance of our findings [11,12,17,45]. Finally, while our data provide strong evidence for transcriptional regulation of *GLI1*, further studies incorporating reporter assays, rescue experiments, and in vivo models will be necessary to fully establish pathway dependency and therapeutic relevance

Despite these limitations, our findings have important translational implications. *EZH2* inhibitors and *LSD1* inhibitors are currently in clinical development, raising the possibility that dual epigenetic targeting strategies could be rapidly translated into therapeutic applications. Notably, the identification of the *STAT3*–*GLI1* axis as a downstream node provides a potential pharmacodynamic biomarker for monitoring treatment response. In addition, given the emerging role of epigenetic therapies in modulating tumor immunogenicity, it will be of interest to determine whether dual *EZH2*/*LSD1* inhibition enhances responses to immunotherapy or targeted agents such as lenvatinib.

In summary, this study defines a cooperative epigenetic mechanism linking *EZH2* and *LSD1* to *STAT3*-dependent *GLI1* transcription in HCC. By disrupting this axis, dual inhibition suppresses multiple tumor-promoting phenotypes, including proliferation, survival, and stemness-associated properties. These findings expand our understanding of how chromatin regulators integrate with oncogenic signaling networks and provide a mechanistic foundation for combinatorial epigenetic therapies in hepatocellular carcinoma.

## 4. Materials and Methods

### 4.1. Bioinformatics Analysis

Gene expression data for *EZH2* and *LSD1* were retrieved from The Cancer Genome Atlas (TCGA) Liver Hepatocellular Carcinoma (LIHC) cohort, and normalized RNA-seq data (log2 FPKM or TPM values) were used. Patients were stratified into high and low expression groups for each gene based on the best cut-off value. Kaplan–Meier survival analysis for overall survival (OS) was performed through the Kaplan–Meier Plotter platform [46]. Log-rank (Mantel–Cox) tests were applied to evaluate statistical significance. Hazard ratios (HRs) and 95% confidence intervals (CIs) were calculated and visualized on the survival curves. GSEA was performed on the TCGA data set using Hallmark gene sets representing major biological pathways, stratified by *EZH2* or *LSD1* expression levels in HCC patients [47].

### 4.2. Cells and Treatment

We used the human hepatocellular carcinoma cell lines HepG2 and Hep3B, obtained from the American Type Culture Collection (ATCC, Manassas, VA, USA), to sample complementary HCC biology. Cells were maintained in Dulbecco’s Modified Eagle’s Medium (DMEM) supplemented with 5% fetal bovine serum (Gibco, Waltham, MA, USA) and 1% penicillin-streptomycin (Thermo Fisher Scientific, Waltham, MA, USA) at 37 °C in a humidified 5% CO_2_ incubator. GSK126 (*EZH2* inhibitor, Cell Signaling Technology, Danvers, MA, USA) and SP2509 (*LSD1* inhibitor, Cell Signaling Technology, Danvers, MA, USA) were purchased from Selleck Chemicals and dissolved in dimethyl sulfoxide (DMSO) to prepare 5 mM stock solutions. For dose-dependent experiments, cells were treated with increasing concentrations (0–40 µM) of each drug or a fixed dual treatment (5 µM each) for 16, 24, 48 h. To inhibit *STAT3* signaling, cells were treated with Stattic (Sigma-Aldrich, St. Louis, MO, USA) [48], a selective *STAT3* inhibitor. Cells were cultured under standard conditions and treated with Stattic at the indicated concentration (0, 2.5, 5, 10 µM) for 24 h. Control cells were treated with an equivalent volume of vehicle (DMSO).

### 4.3. Small Interfering RNA Transfection

Cells were transfected with small interfering (si) RNAs targeting *EZH2* (siEZH2 from Sigma Aldrich, siRNA ID: SASI_Hs02_00337644), *LSD1* (siLSD1, siRNA ID: SASI_Hs01_00213079), or both, along with scrambled siRNA-transfected cells as a control (siRNA Universal Negative control: SIC001). After 48 h of transfection, cells were harvested and subjected to downstream analysis.

### 4.4. Cell Viability, IC_50_ Determination, and Synergy Score

Cell proliferation and viability were assessed using the MTT assay (Sigma-Aldrich, Burlington, MA, USA). Cells were seeded into 96-well plates (5 × 10^3^ cells/well) and allowed to adhere overnight before drug treatment. After 48 h, MTT reagent (0.5 mg/mL) was added to each well and incubated for 3 h at 37 °C. The medium was removed, and formazan crystals were dissolved in 100 µL of DMSO. Absorbance was measured at 570 nm using a microplate reader. IC_50_ values were calculated by nonlinear regression using a log vs. response curve. All the synergy scores were determined by using the SynergyFinder software (Version 3.10.3) [26].

### 4.5. Wound Healing Assay

For migration, HepG2 and Hep3B were seeded into 6 well plates and grown to 100% confluence. A linear wound was created using a 200 µL pipette tip, followed by washing with PBS to remove debris. Cells were then cultured in 1% FBS DMEM to minimize proliferation effects and treated with GSK126 (1, 2.5, 5 µM), SP2509 (1, 2.5, 5 µM), or the dual treatment for 48 h. Wound areas were photographed at 0 and 48 h using an inverted phase contrast microscope (×100 magnification). The relative wound closure was calculated using ImageJ software (version 1.54) by measuring the width of the wound at both time points. Multiple width measurements were taken at several points along each wound, and the values were averaged to obtain the final width for that well.

### 4.6. Invasion Assay

Cell invasion was assessed using Transwell chambers fitted with 8.0 µm pore size inserts (Corning, NY, USA) pre-coated with growth factor–reduced Matrigel (BD Biosciences, San Jose, CA, USA) diluted 1:9 in serum-free DMEM. Following 48 h treatment with GSK126 (2.5, 5 µM), SP2509 (2.5, 5 µM), or the dual treatment, HepG2 and Hep3B cells were collected, resuspended in serum-free medium, and seeded into the upper chamber at a density of 2 × 10^4^ cells per well in 200 µL. The lower chamber contained 600 µL of DMEM supplemented with 10% FBS as a chemoattractant. After 24 h of incubation at 37 °C in a humidified incubator with 5% CO_2_, non-invading cells on the upper surface of the membrane were gently removed with cotton swabs. Invaded cells on the underside of the membrane were fixed in methanol and stained with 0.5% crystal violet. Images were captured using an inverted microscope, and invasion was quantified by counting the number of stained cells in multiple randomly selected fields per insert.

### 4.7. Cell Cycle Analysis by PI Staining (FACS)

To assess the impact of dual inhibition on cell cycle progression, flow cytometric analysis was performed following propidium iodide (PI) staining. HepG2 and Hep3B cells were treated with vehicle, GSK126 (5 µM), SP2509 (5 µM), or the dual treatment for 48 h, then harvested and fixed overnight in 70% ethanol at −20 °C. Fixed cells were washed with PBS, incubated with RNase A (100 µg/mL), and stained with PI (50 µg/mL) for 30 min at room temperature in the dark. DNA content was analyzed using a FACS Canto II flow cytometer (BD Biosciences, San Jose, CA, USA), and cell cycle distribution was quantified using FlowJo software (v10).

### 4.8. TUNEL Assay and Imaging

Apoptotic DNA fragmentation was evaluated using the In Situ Cell Death Detection Kit, Fluorescein (Roche, Basel, Switzerland), according to the manufacturer’s instructions. HepG2 and Hep3B cells were seeded on sterile glass coverslips and treated with GSK126 (5 µM), SP2509 (5 µM), or both for 48 h. Cells were then fixed in 4% paraformaldehyde (Cell Signaling Technology, Danvers, MA, USA)), permeabilized with 0.1% Triton X-100 (Sigma-Aldrich, Burlington, MA, USA) in PBS, and incubated with TUNEL reaction mixture for 1 h at 37 °C in a humidified dark chamber. Nuclei were counterstained with DAPI, and fluorescence images were captured using a fluorescence microscope (×200 magnification). The number of TUNEL-positive nuclei was quantified in six to eight randomly selected fields per sample and expressed as a percentage of the total number of nuclei in those fields.

### 4.9. Quantitative Real-Time PCR (qRT-PCR)

For gene expression analyses, total RNA was isolated from HepG2 and Hep3B using RNAZoL (Sigma-Aldrich, Burlington, MA, USA). Total RNA (1 µg) was reverse-transcribed with cDNA Reverse Transcription Kit (Thermo Fisher Scientific, Waltham, MA, USA) and cDNA was synthesized from 1 µg of RNA using the iScript cDNA Synthesis Kit (Bio-Rad, Hercules, CA, USA). qRT-PCR was carried out using SYBR Green Supermix (Bio-Rad, Hercules, CA, USA) on a QuantStudio™ 5 Real-Time PCR System (Thermo Fisher Scientific, Waltham, MA, USA).

Primer sequences specific to EZH2, LSD1, STAT3, GLI1, C-MYC, Cyclin D1, Vimentin, MMP9, β-catenin, Sp1, and β-Actin were used (Supplementary Table S1). β-Actin served as the internal control, and relative gene expression was calculated using the 2^−ΔΔCt^ method. Each reaction was performed in technical triplicates, and experiments were independently repeated at least three times.

### 4.10. Caspase 3/7 Activity Assay

To assess apoptosis, the Caspase-Glo 3/7 Assay Kit (Promega, Fitchburg, WI, USA) was used according to the manufacturer’s protocol. Cells were plated in white 96-well plates (1 × 10^4^ cells/well) and treated with inhibitors for 24 h. An equal volume of Caspase-Glo reagent was added directly to each well and incubated at room temperature for 1 h in the dark. Data were normalized to untreated controls and plotted as relative caspase activity.

### 4.11. Tumor Sphere Formation Assay

To evaluate self-renewal and stem-like characteristics, cells were cultured under non-adherent, serum-free conditions in ultra-low attachment 6 well plates (Corning, NY, USA). Cells (1 × 10^4^ per well) were suspended in DMEM/F12 supplemented with B27 supplement (1:50, Gibco), EGF (20 ng/mL), and bFGF (20 ng/mL). GSK126 and SP2509 (2.5, 5 µM) were added on day 0. After 14 days, tumor spheres > 50 µm were counted using an inverted microscope, and representative images were taken. Sphere numbers were quantified from 4 to 6 independent replicate wells to minimize experimental variability. Cell viability was assessed in 6 independent replicate wells using the Promega^®^ Cell Titer-Glo^®^ 3D assay.

### 4.12. Immunoblotting

Total proteins were extracted using RIPA buffer containing protease and phosphatase inhibitors (Thermo Fisher, Waltham, MA, USA). Protein concentrations were determined using the BCA assay. Equal amounts of protein were separated by SDS-PAGE and transferred to PVDF membranes (Millipore, Burlington, MA, USA). Membranes were blocked with 5% BSA or non-fat milk and incubated overnight at 4 °C with primary antibodies against: EZH2, LSD1, PCNA, Cyclin D1, Vimentin, MMP9, β-catenin, Sp1, Pro-Caspase 3, Pro-Caspase 7, STAT3, GLI1, C-MYC, H3K27ME3, H3K4ME2, H3 (all from Cell Signaling Technology, Danvers, MA, USA or Proteintech, Rosemont, IL, USA) (Supplementary Table S2). β-Actin was used as loading control. After washing, membranes were incubated with HRP-conjugated secondary antibodies, and proteins were detected using ECL substrate and visualized with ChemiDoc Imaging System. All immunoblotting experiments were performed independently at least two to three times. Band intensities were quantified using densitometric analysis, and the corresponding quantification data for each figure are provided (Supplementary Figures S1, S2, S5, S6, S7, S9, S11 and S12). All original immunoblotting images are included (Supplementary Figures S13 and S14).

### 4.13. ChIP Assay

Chromatin immunoprecipitation (ChIP) assays were performed using a ChIP assay kit (CUT & RUN Fast, EPIGENTEK, Farmingdale, NY, USA). Briefly, cells were crosslinked with 0.1% formaldehyde at room temperature for 2 min, and the reaction was quenched by the addition of 125 mM glycine. Cells were harvested and lysed in ChIP lysis buffer supplemented with protease inhibitors. Equal amounts of sheared chromatin were incubated overnight at 4 °C with antibodies against STAT3, EZH2, LSD1, H3K27ME3, H3K4ME2, RNA polymerase II and normal IgG as a negative control. Immune complexes were captured using magnetic beads. Bound chromatin was eluted, and crosslinks were reversed by incubation at 60 °C, 15 min, followed by proteinase K. Purified DNA was recovered using a DNA purification magnetic bead and analyzed by quantitative PCR using GLI1 gene promoter primer set [5′ Forward (−1021): GGA TAA CTA GCC TGG GTC AAA G, 3′ Reverse (−752): GCC ATC GTG ACC GGA TAT AAG]. ChIP enrichment was calculated as a percentage of input DNA or normalized to IgG control.

### 4.14. Statistical Analysis

All experiments were independently repeated at least three times. Statistical analysis was performed using GraphPad Prism 10. Comparisons among three or more groups were performed using one-way ANOVA. Two-way ANOVA was used when two independent variables were involved. All quantification results are shown as the mean ± SD (Statistical significance was defined as * *p* < 0.05, ** *p* < 0.01, *** *p* < 0.001, **** *p* < 0.0001).

## 5. Conclusions

In conclusion, this study demonstrates that *EZH2* and *LSD1* are not only independent oncogenic drivers in hepatocellular carcinoma (HCC), but also function cooperatively as critical epigenetic regulators that sustain tumor progression and aggressive phenotypes. Analysis of the TCGA-LIHC cohort revealed that high expression of *EZH2* and *LSD1* was each significantly associated with poor overall survival, and patients with co-elevated expression of both factors exhibited the worst prognosis, supporting their clinical relevance as cooperative prognostic markers. Functionally, dual inhibition of *EZH2* and *LSD1* effectively suppressed HCC cell proliferation, disrupted cell cycle progression, and induced caspase-dependent apoptosis. In addition, combined targeting markedly reduced migration, invasion, and tumor sphere formation, indicating strong suppression of metastatic potential and cancer stem-like properties. Mechanistically, *EZH2* and *LSD1* were found to cooperate with *STAT3* at the *GLI1* promoter to sustain Sonic Hedgehog (SHH) signaling, a key pathway involved in HCC progression and stemness maintenance. Dual inhibition significantly reduced the occupancy of *EZH2*, *LSD1*, *STAT3*, and RNA polymerase II at the *GLI1* promoter, resulting in transcriptional repression of *GLI1* and attenuation of its downstream oncogenic targets. Suppression of the *STAT3*–*GLI1* axis emerged as a central mechanism underlying SHH pathway inhibition and the resulting antitumor effects. Collectively, these findings establish dual targeting of *EZH2* and *LSD1* as a mechanistically defined therapeutic strategy that disrupts a core oncogenic signaling network in HCC. This study provides strong preclinical evidence supporting the translational potential of combinatorial epigenetic therapy for improving treatment outcomes in hepatocellular carcinoma.

## Data Availability

The original contributions presented in this study are included in the article/Supplementary Material. Further inquiries can be directed to the corresponding author.

## Supplementary Materials

The following supporting information can be downloaded at: https://www.mdpi.com/article/10.3390/ijms27093886/s1.

## Abbreviations

The following abbreviations are used in this manuscript:

HCC	Hepatocellular Carcinoma
ATCC	American Type Culture Collection
TCGA	The Cancer Genome Atlas
LIHC	Liver Hepatocellular Carcinoma
EZH2	Enhancer of Zeste Homolog 2
LSD1	Lysine-Specific Demethylase 1
GSEA	Gene Set Enrichment Analysis
CCND1	Cyclin D1
PCNA	Proliferating cell nuclear antigen
SHH	Sonic Hedgehog
STAT3	Signal transducer and activator of transcription 3
GLI1	Glioma-associated oncogene homolog 1
PRC2	Polycomb Repressive Complex 2
FAD	Flavin Adenine Dinucleotide
H3K27ME3	Histone H3 lysine 27 trimethylation
H3K4ME2	Histone H3 lysine 4 dimethylation
H3v	Histone H3
HRs	Hazard Ratios
CIs	Confidence Intervals
DMEM	Dulbecco’s Modified Eagle’s Medium
DMSO	Di-Methyl Sulfoxide
MMP9	Matrix Metalloproteinase-9
VIM	Vimentin
SP1	Specificity Protein 1
bFGF	basic Fibroblast Growth Factor
EGF	Epidermal Growth Factor
PDX	Patient-Derived Xenograft
ES	Enrichment score
VEGF-A	Vascular Endothelial Growth Factor A
OS	Overall survival
siRNA	Small interfering RNA
qRT-PCR	quantitative real-time PCR
TUNEL	Terminal deoxynucleotidyl transferase dUTP nick end labeling
FBS	Fetal Bovine Serum
IgG	Immunoglobulin G
ChIP	Chromatin Immunoprecipitation
CUT&RUN	Cleavage Under Targets and Release Using Nuclease
