# Supplementary material for: Dual Targeting of EZH2 and LSD1 Suppresses Hepatocellular Carcinoma via Disruption of Sonic Hedgehog Signaling
Source: Int J Mol Sci. 2026 Apr 27;27(9):3886. doi: 10.3390/ijms27093886 (PMC13163952; doi:10.3390/ijms27093886)

Supplementary Table 1. Human Primer Sequence

Primer Name	Sequence
EZH2 Human Forward Sequence	GACCTCTGTCTTACTTGTGGAGC
EZH2 Human Reverse Sequence	CGTCAGATGGTGCCAGCAATAG
KDM1A(LSD1) Human Forward Sequence	TCAGGAGTTGGAAGCGAATCCC
KDM1A(LSD1) Human Reverse Sequence	GTTGAGAGAGGTGTGGCATTAGC
PCNA Human Forward Sequence	CAAGTAATGTCGATAAAGAGGAGG
PCNA Human Reverse Sequence	GTGTCACCGTTGAAGAGAGTGG
Vimentin (VIM) Human Forward Sequence	TCTACACCGACAACTCCATCCG
Vimentin (VIM) Human Reverse Sequence	TCTGGCATTTTGGAGAGGAAGTG
MMP9 Human Forward Primer	TGTACCGCTATGGTTACACTCG
MMP9 Human Reverse Primer	GGCAGGGACAGTTGCTTCT
CTNNB1 β-catenin Human Forward Sequence	CACAAGCAGAGTGCTGAAGGTG
CTNNB1 β-catenin Human Reverse Sequence	GATTCCTGAGAGTCCAAAGACAG
SP1 Human Forward Sequence	ACGCTTCACACGTTCGGATGAG
SP1 Human Reverse Sequence	TGACAGGTGGTCACTCCTCATG
β-Actin Human Forward Sequence	CACCATTGGCAATGAGCGGTTC
β-Actin Human Reverse Sequence	AGGTCTTTGCGGATGTCCACGT
GLI1 Human Forward Sequence	AGCCTTCAGCAATGCCAGTGAC
GLI1 Human Reverse Sequence	GTCAGGACCATGCACTGTCTTG
C-MYC Human Forward Sequence	CCTGGTGCTCCATGAGGAGAC
C-MYC Human Reverse Sequence	CAGACTCTGACCTTTTGCCAGG
Cyclin D1 Human Forward Sequence	TCTACACCGACAACTCCATCCG
Cyclin D1 Human Reverse Sequence	TCTGGCATTTTGGAGAGGAAGTG
STAT3 Human Forward Sequence	CTTTGAGACCGAGGTGTATCACC
STAT3 Human Reverse Sequence	GGTCAGCATGTTGTACCACAGG

Supplementary Table 2. Antibodies for Immunoblotting

Antibody Name	Company	Cat No
EZH2(D2C9)	Cell Signaling Technology	#5246
LSD1	Cell Signaling Technology	#2139
β-catenin	Cell Signaling Technology	#8480
PCNA	Proteintech	10205-2-AP
Cyclin D1	Proteintech	60186-1-Ig
Vimentin	Proteintech	10366-1-AP
MMP9	Proteintech	10366-1-AP
Sp1	Proteintech	21962-1-AP
Caspase-3	Proteintech	19677-1-AP
Caspase-7	Proteintech	27155-1-AP
β-Actin	Proteintech	66009-1-Ig
H3	Proteintech	17168-1-AP
GLI1	Proteintech	66905-1-Ig
STAT3	Cell Signaling Technology	#12640
C-MYC	Cell Signaling Technology	#5605
H3K27ME3	Cell Signaling Technology	#8173
H3K4ME2	Cell Signaling Technology	#9725

Supplementary Table 3

Synergy Score Summary Table HepG2				
Block ID	ZIP	Loewe	HSA	Bliss
1	1.07	8.98	11.17	1.77

Supplementary Table 4

Sensitivity Score Summary Table HepG2			
Block ID	RI_1	RI_2	CSS
1	65.27	40.16	92.09

Supplementary Table 5

Synergy Score Summary Table Hep3B				
Block ID	ZIP	Loewe	HSA	Bliss
1	9.55	8.66	16.34	8.17

Supplementary Table 6

Sensitivity Score Summary Table Hep3B			
Block ID	RI_1	RI_2	CSS
1	37.8	42.92	84.35

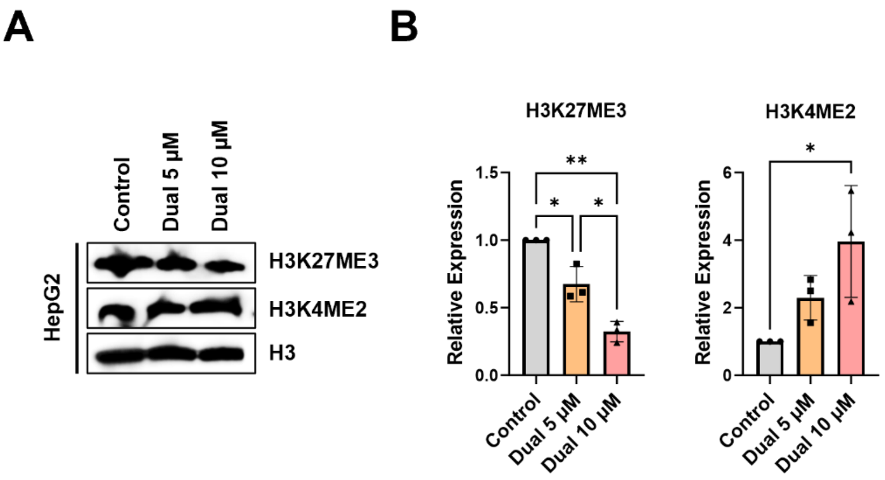

Supplementary Figure 1. Effects of Dual inhibitor treatment on the downstream target histone modification.

A. Immunoblotting showed that dual inhibitor treatment of HepG2 for 24 hours reduced the level of H3K27ME3, an *EZH2* substrate, while increasing H4K4ME2, an *LSD1* substrate, in a dose-dependent manner. **B**. Quantification of H3K27ME3 and H3K4ME2 bands from three independent experiments. Protein expression levels were normalized to the respective loading controls and expressed relative to the control group. Data are presented as mean ± SD of three independent experiments. All quantification results are shown as the mean ± SD (*$p < 0.05$, **$p < 0.01$ by two-way ANOVA).

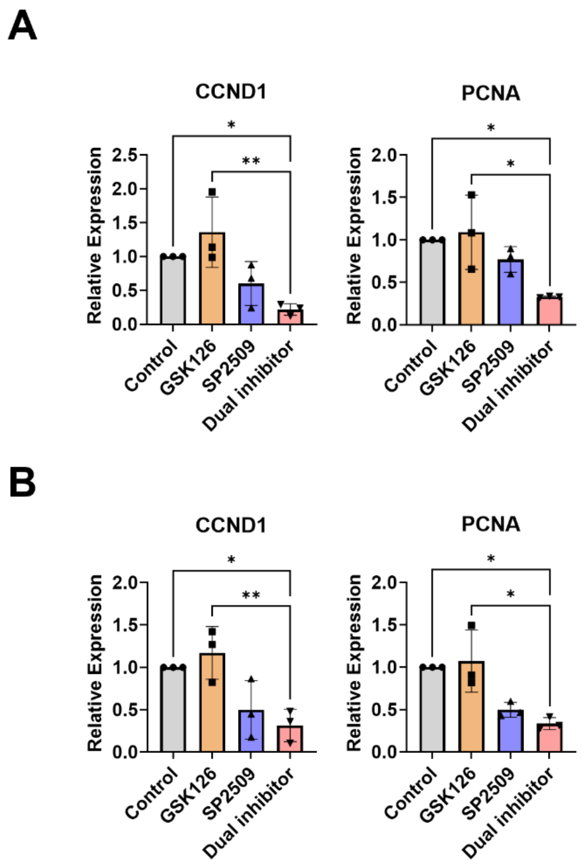

Supplementary Figure 2. Dual inhibition of *EZH2* and *LSD1* modulates cell cycle associated signaling in HCC cells.

A, **B**. HepG2 (**A**) and Hep3B (**B**) were treated with *EZH2* (GSK126, 5 μM) and *LSD1* (SP2509, 5 μM) single inhibitors or dual treatment as indicated, and the expression levels of cell cycle-associated regulatory proteins were analyzed. Cell lysates were subjected to SDS-PAGE followed by immunoblotting to assess the abundance of CCND1 and PCNA. Protein expression levels were normalized to the respective loading controls and expressed relative to the control group. Immunoblotting shows decreased expression of cell proliferation markers CCND1 and PCNA following single or Dual treatments. All Data represent mean ± SD from three independent experiments. All quantification results are shown as the mean ± SD (*$p < 0.05$, **$p < 0.01$ by two-way ANOVA).

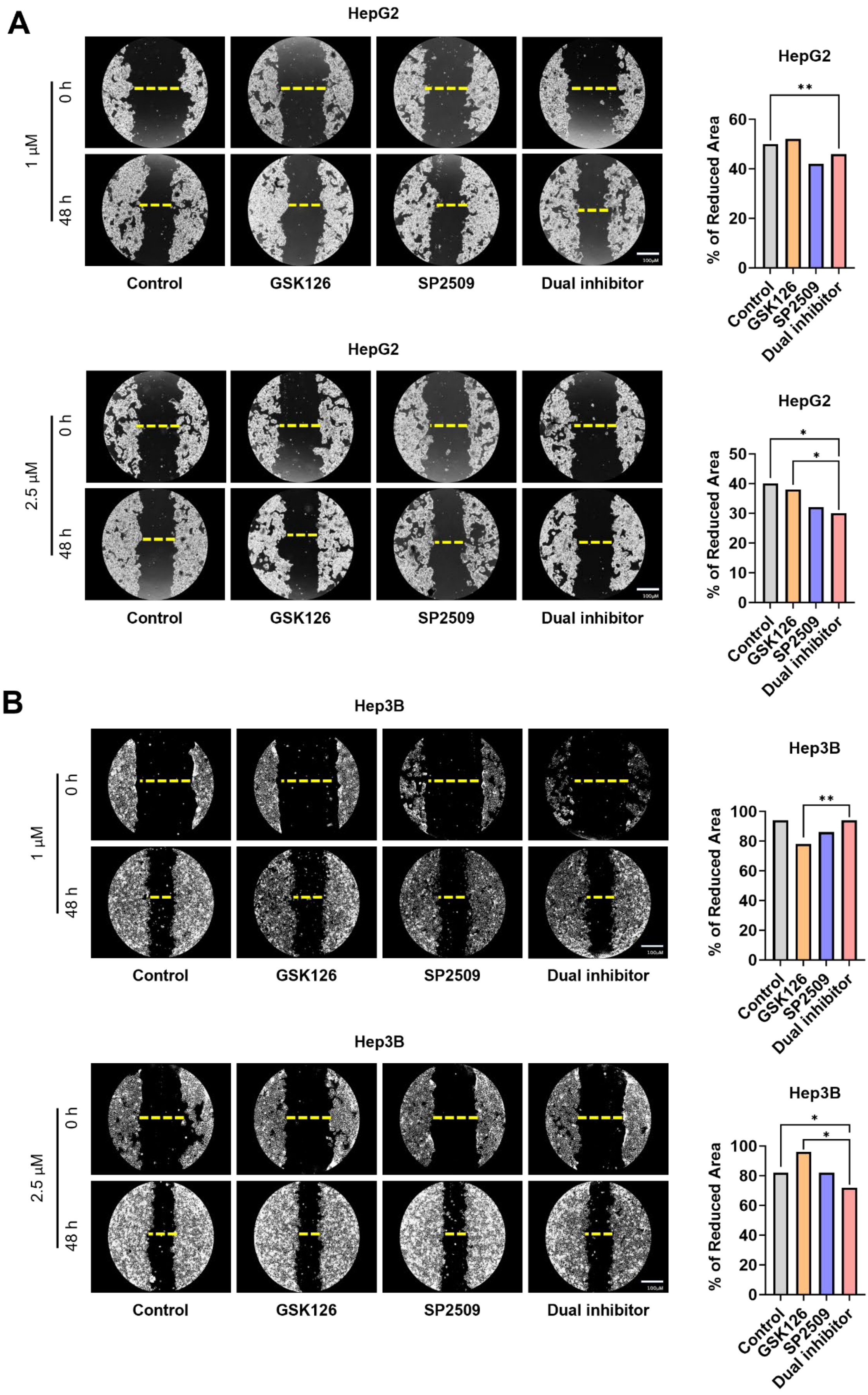
A
HepG2
1 µM
0 h
48 h
Control
GSK126
SP2509
Dual inhibitor
HepG2
% of Reduced Area
**
2.5 µM
*
B
Hep3B
100µM

Supplementary Figure 3. Effects of Dual inhibition on HCC cell migration.

A, **B**. Representative images of scratch wound healing assays and % of reduced area in HepG2 and Hep3B cells treated with GSK126 1, 2.5 μM, SP2509 1, 2.5 μM, or Dual inhibition for 48 h. Wound width was measured at 0 and 48 h and expressed as percent closure (x 20, Scale bar, 100 μm). All Data represent mean ± SD from three independent experiments. All quantification results are shown as the mean ± SD (*$p < 0.05$, **$p < 0.01$ by one-way ANOVA).

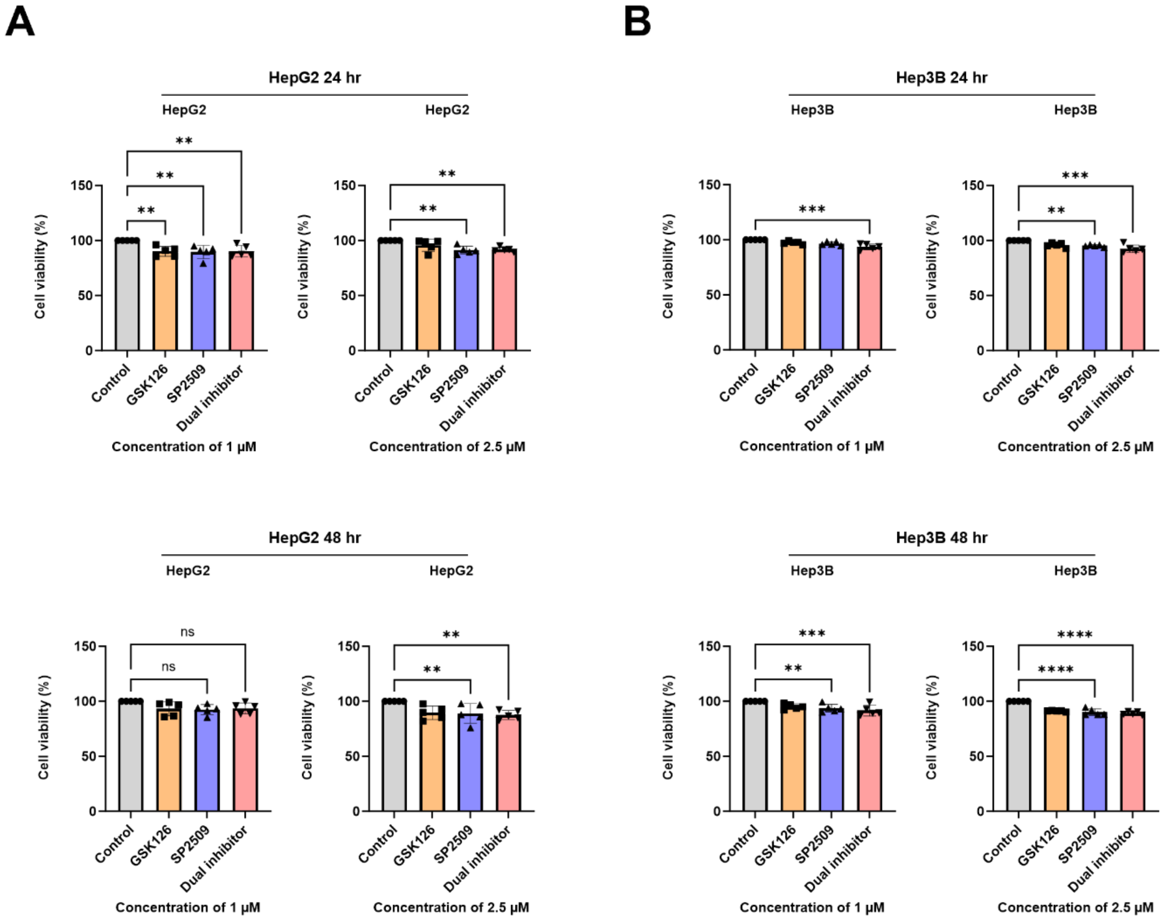

Supplementary Figure 4. Effects of dual inhibition on HCC cell viability.

A, **B**. MTT assay for the *EZH2* inhibitor GSK126 and the *LSD1* inhibitor SP2509 and Dual inhibitor with 1, 2.5 μM concentration in HepG2 and Hep3B cells after 24, 48 h of treatment. All Data represent mean ± SD from three independent experiments. All quantification results are shown as the mean ± SD $^{**}p < 0.01$, $^{***}p < 0.001$, $^{****}p < 0.0001$ by two-way ANOVA).

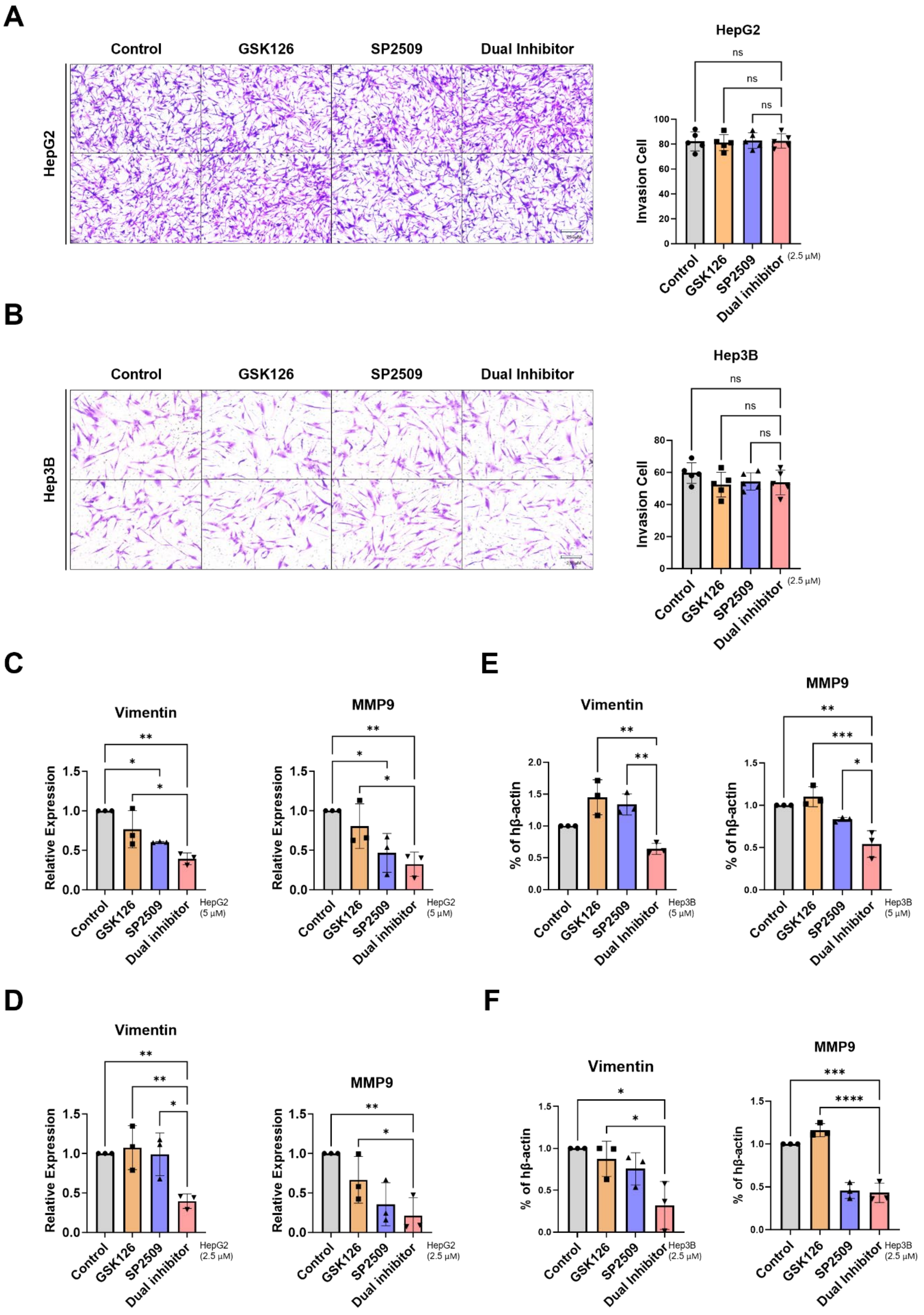

Supplementary Figure 5. Dual inhibition of *EZH2* and *LSD1* suppresses invasion and metastasis of HCC cells.

A, **B**. HepG2 and Hep3B cells were treated with GSK126 2.5 μM, SP2509 2.5 μM, or Dual treatment for 48 h, followed by invasion assays using Matrigel-coated Trans-well membrane for 24 h from the four group. (magnification, x 200, scale bar, 250 μm). **C**-**F**. Relative protein expression levels of EMT-related migration markers vimentin and MMP9 in HepG2 and Hep3B cells after with GSK126 2.5, 5 μM, SP2509 2.5, 5 μM, or Dual treatment for 48 h. The levels of inactive vimentin and MMP9 were quantified and normalized to the control group. All Data represent mean ± SD from three independent experiments. All quantification results are shown as the mean ± SD (*$p < 0.05$, **$p < 0.01$, ***$p < 0.001$, ****$p < 0.0001$ by two-way ANOVA).

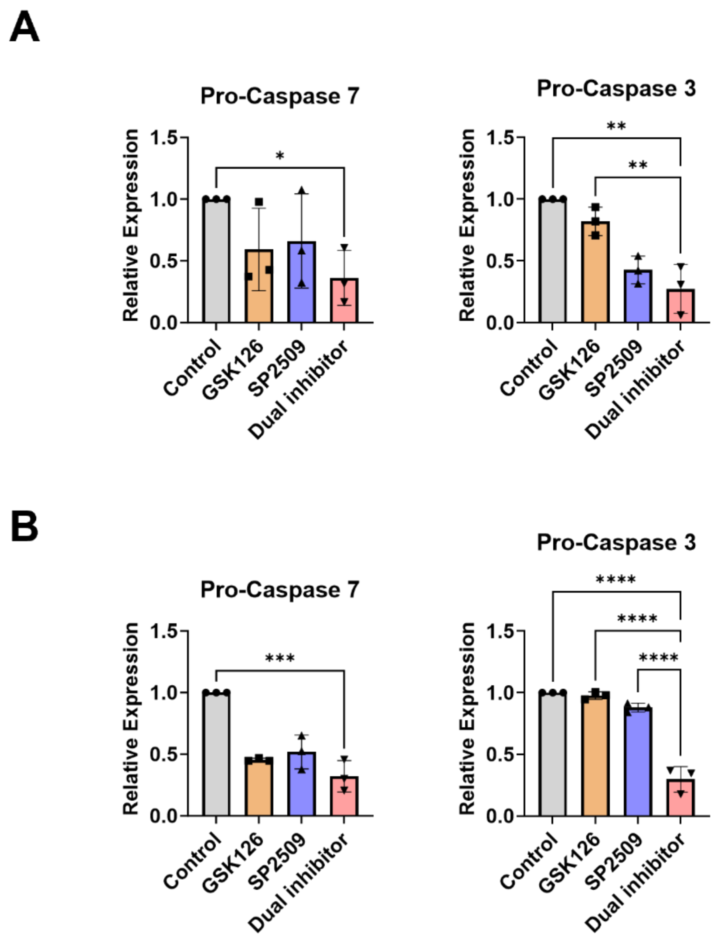

Supplementary Figure 6. Modulation of apoptosis-related protein levels following *EZH2* and *LSD1* Dual inhibition.

A, **B**. Relative protein expression levels of apoptosis-related markers in HepG2 (**A**) and Hep3B (**B**) cells. Cells were treated with *EZH2* inhibitor (GSK126, 5 μM), *LSD1* inhibitor (SP2509, 5 μM), Dual inhibitor for 48 h. The levels of inactive Pro-Caspase 3, 7 were quantified and normalized to the control group. Dual inhibition of *EZH2* and *LSD1* led to a significant reduction in the pro-forms of these markers, indicating an enhanced induction of the apoptotic cascade compared to single-agent treatments. The levels of inactive vimentin and Pro-Caspase 3, 7 were quantified and normalized to the control group. Data are presented as mean ± SD of three independent experiments. All quantification results are shown as the mean ± SD (*p < 0.05, **p < 0.01, ***p < 0.001, ****p < 0.0001 by two-way ANOVA).

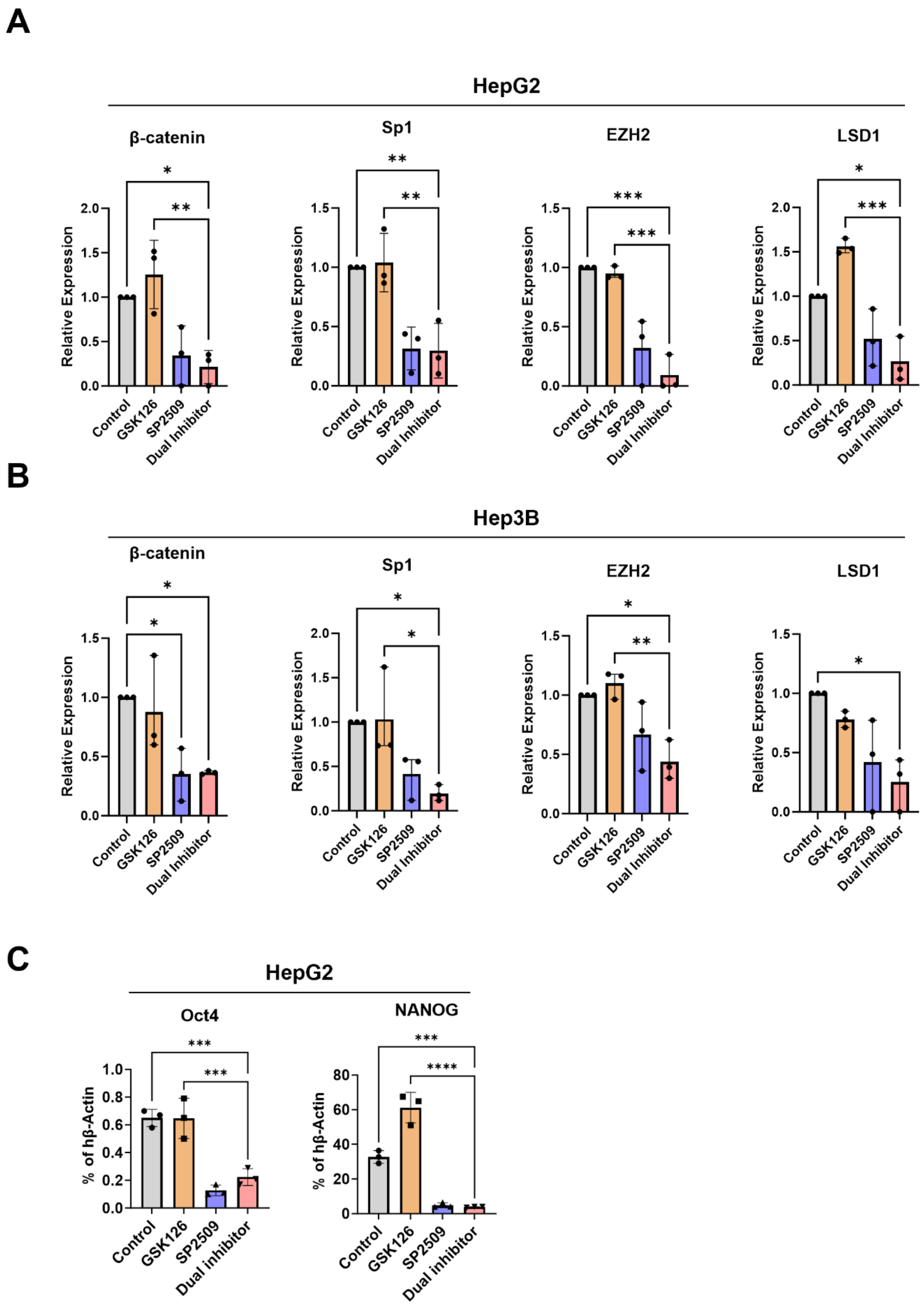

Supplementary Figure 7. Dual inhibition of *EZH2* and *LSD1* suppresses attenuates stemness-associated signatures in HCC cells.

A, **B**. HepG2 (**A**) and Hep3B (**B**) were treated with *EZH2* inhibitor (GSK126, 5 μM), LSD1 inhibitor (SP2509, 5 μM), or Dual Inhibitor. The expression levels of *β*-catenin, Sp1, EZH2, and LSD1 were significantly downregulated in the Dual inhibition group compared to single treatments in HCC cell lines. **C**. HepG2 was treated with *EZH2* inhibitor, *LSD1* inhibitor or Dual inhibitor. The mRNA expression levels of *NANOG* and *Oct4* were significantly downregulated in the Dual inhibitor group compared to the control group. Data are presented as mean ± SD of three independent experiments. All quantification results are shown as the mean ± SD (*$p < 0.05$, **$p < 0.01$, ***$p < 0.001$, ****$p < 0.0001$ by two-way ANOVA).

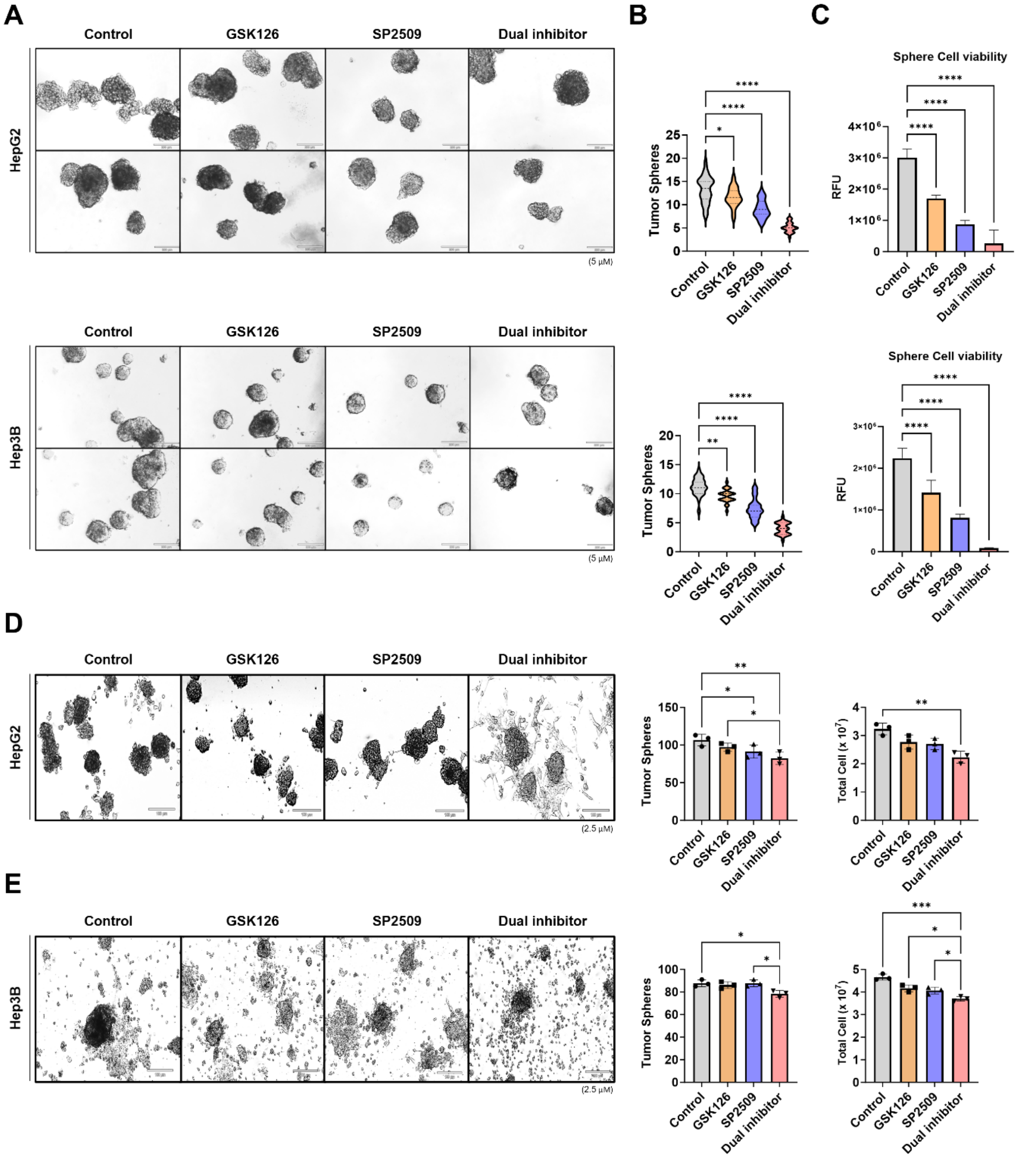

Supplementary Figure 8. Disruption of pre-established Tumor Sphere by dual inhibition of *EZH2* and *LSD1*.

A, **B**. Representative images and tumor spheres quantitative analysis results of sphere formation in HepG2 and Hep3B cells cultured for 14 days, treated with 5 µM GSK126, 5 µM SP2509, or dual inhibitors. Cells were seeded in 96-well plates at a density of 5 x 10^2 cells per well. Sphere numbers per well were counted after treatment (magnification, x 200, scale bar, 200 µm). **C**. Sphere Cell viability was assessed in 6 independent replicate wells using the Promega® Cell Titer-Glo® 3D assay. **D**, **E**. Representative images and quantification of tumor spheres formed by HepG2 and Hep3B cells cultured with GSK126 2.5 µM, SP2509 2.5 µM, or dual inhibitor treatment (magnification, x 200, scale bar, 100 µm). Cells were seeded in 96-well plates at a density of 5 x 10^2 cells per well. Sphere numbers per well were counted after

14 days. Data are presented as mean ± SD of three independent experiments. All quantification results are shown as the mean ± SD (*$p < 0.05$, **$p < 0.01$, ***$p < 0.001$, ****$p < 0.0001$ by two-way ANOVA).

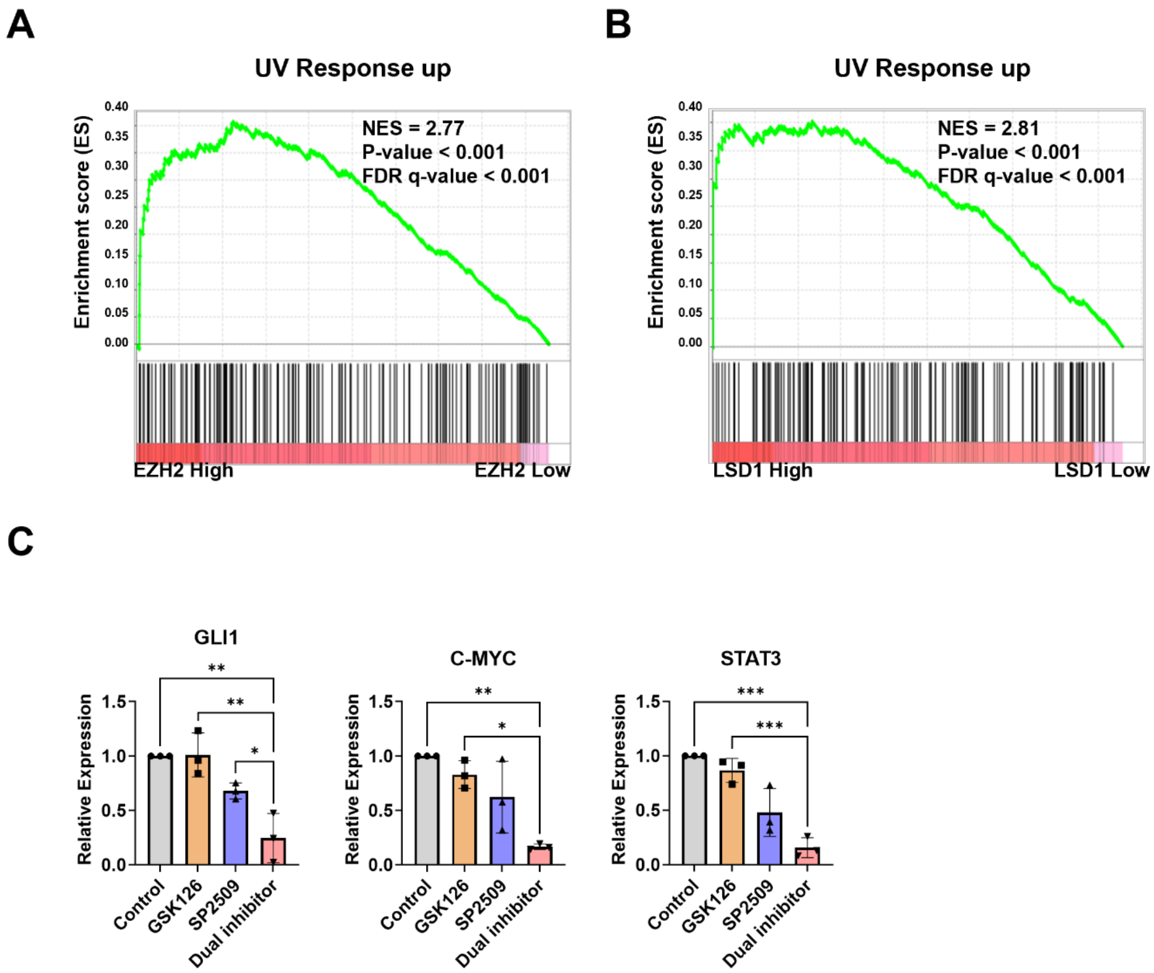

Supplementary Figure 9. Dual inhibition of *EZH2* and *LSD1* suppresses UV Response gene via attenuation of the *STAT3–GLI1* axis in HCC.

A, **B** Gene Set Enrichment Analysis (GSEA) plots showing the enrichment of UV Response up gene sets in $\mathrm{EZH2}^{\mathrm{High/Low}}$ and $\mathrm{LSD1}^{\mathrm{High/Low}}$ groups. The GSEA analysis was performed with the Hallmark gene signature. **C**. Relative protein expression levels of key *STAT3-GLI1* signaling pathway including *GLI1*, C-MYC, *STAT3* in HepG2 cells. Cells were treated with *EZH2* inhibitor (GSK126, 5 µM), *LSD1* inhibitor (SP2509, 5 µM), Dual inhibitor for 48 h. The levels of inactive *GLI1*, C-MYC, *STAT3* were quantified and normalized to the control group. Data are presented as mean ± SD of three independent experiments. All quantification results are shown as the mean ± SD (**p* < 0.05, ***p* < 0.01, ***p < 0.001 by two-way ANOVA).

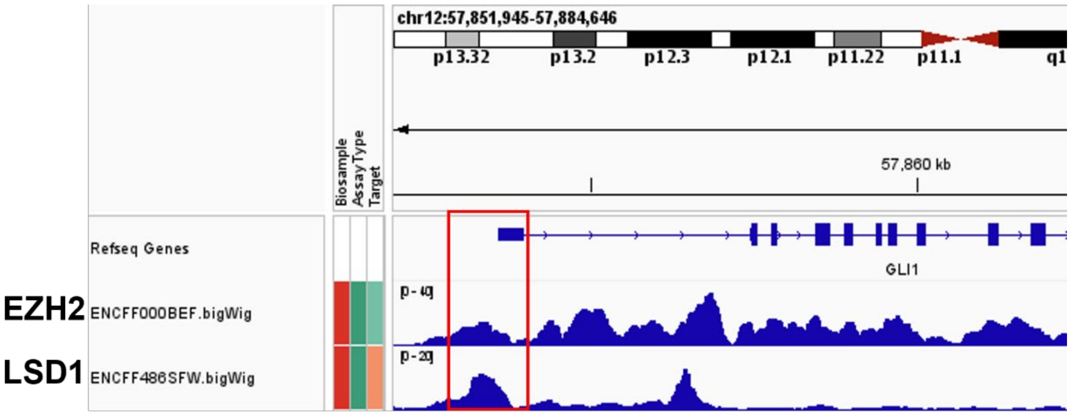

Supplementary Figure 10. Colocalization of *EZH2* and *LSD1* at the *GLI1* promoter region.

Integrated Genomics Viewer (IGV) tracks displaying ChIP-seq signal enrichment for *EZH2* and *LSD1* across the *GLI1* locus. Genomic coordinates and RefSeq gene annotations are shown above the signal tracks. Both *EZH2* and *LSD1* exhibit overlapping peaks at the promoter region of *GLI1*, indicating co-occupancy at this regulatory site (red box). Signal intensity represents normalized read density from bigWig tracks. These data support coordinated epigenetic regulation of *GLI1* by *EZH2* and *LSD1*. *EZH2* and *LSD1* signal tracks were derived from ENCODE Project datasets (file accession numbers: ENCFF000BEF and ENCFF486SFW, respectively).

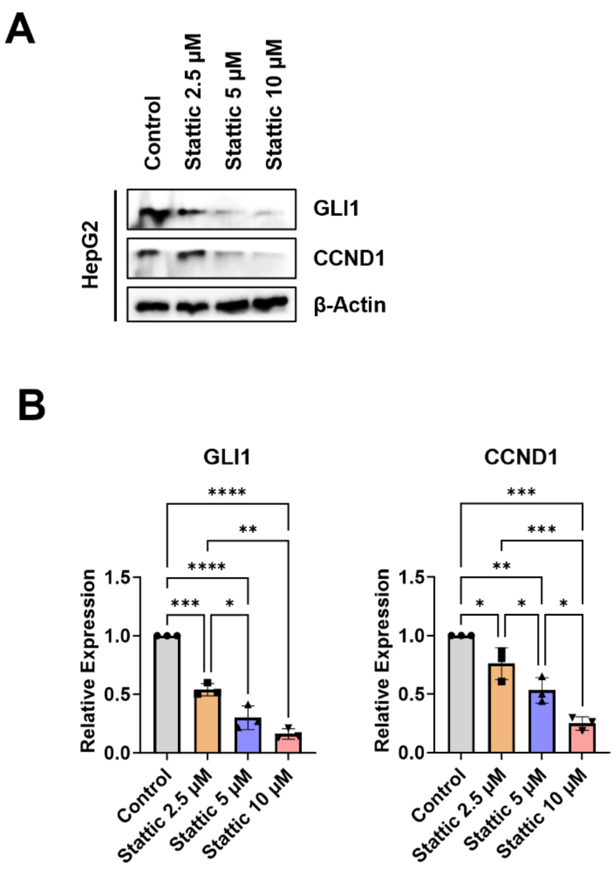

Supplementary Figure 11. Reduced expression of *GLI1* by STATTIC, a selective *STAT3* inhibitor.

A. Immunoblot analysis of HepG2 cells treated with the *STAT3* inhibitor STATTIC (0, 2.5, 5, and 10 µM for 48 h), demonstrating dose-dependent suppression of *GLI1* signaling. **B**. Relative protein expression levels of *GLI1* and CCND1 in HepG2 cells. The levels of inactive *GLI1* and CCND1 were quantified and normalized to the control group. Data are presented as mean ± SD of three independent experiments. All quantification results are shown as the mean ± SD (**p* < 0.05, ***p* < 0.01, ***p < 0.001, ****p < 0.0001 by two-way ANOVA).

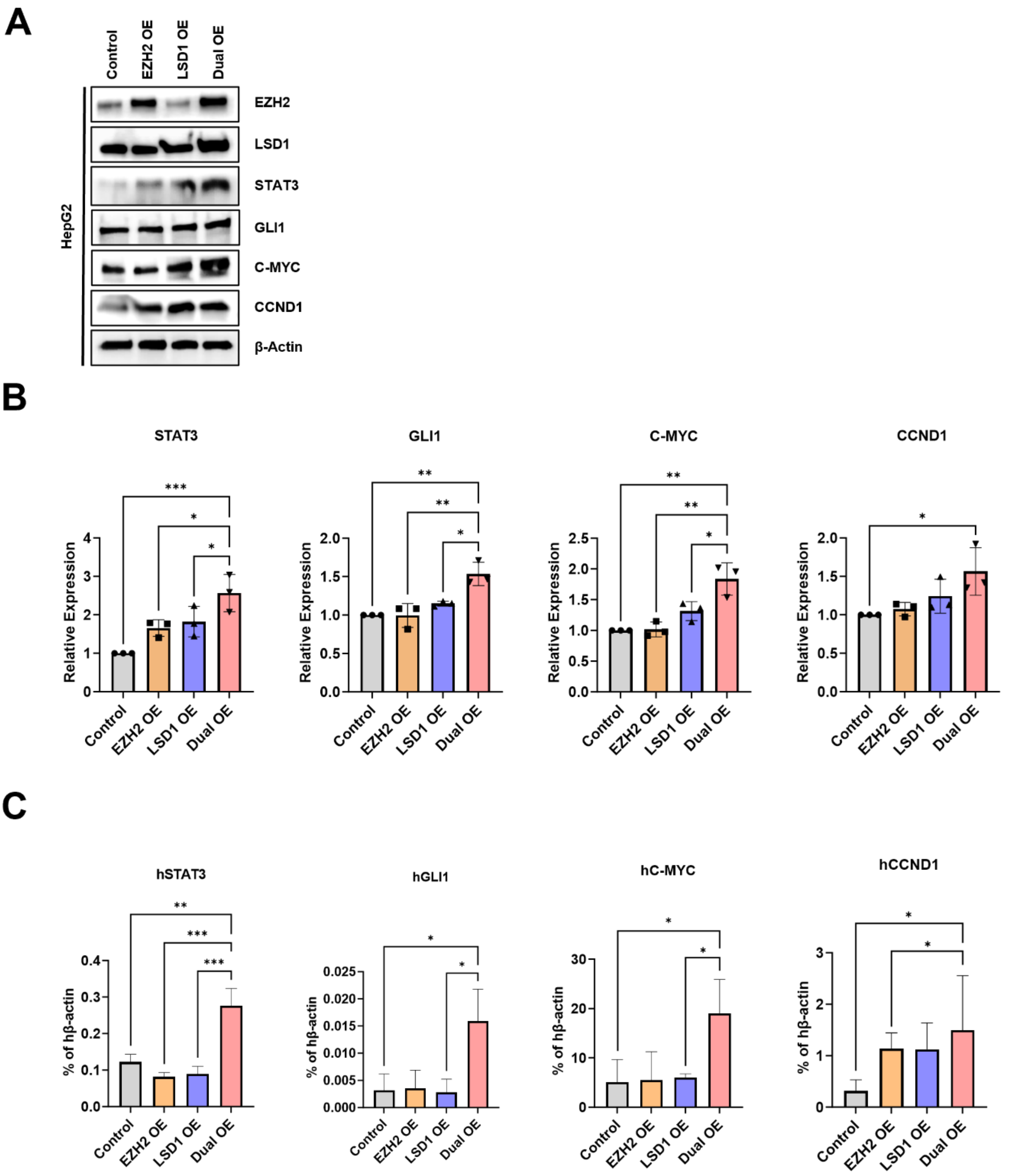

Supplementary Figure 12. Overexpression of *EZH2* and *LSD1* increased *STAT3*, *GLI1*, C-MYC, and Cyclin D1 expression at both mRNA and protein levels.

A-**C**. HepG2 cells were transfected with expression vectors for *EZH2*, *LSD1*, or Dual Over expression. Dual OE of these factors led to a significant increase in the protein(**A**) and relative protein expression levels(**B**) mRNA(**C**) of *STAT3*, *GLI1*, C-MYC and CCND1 in HepG2 cells. The levels of inactive *STAT3*, *GLI1*, C-MYC and CCND1 were quantified and normalized to the control group. Data are presented as mean ± SD of three independent experiments. All quantification results are shown as the mean ± SD (*$p < 0.05$, **$p < 0.01$, ***p < 0.001 by two-way ANOVA).

Supplementary Figure 13

Figure 2E

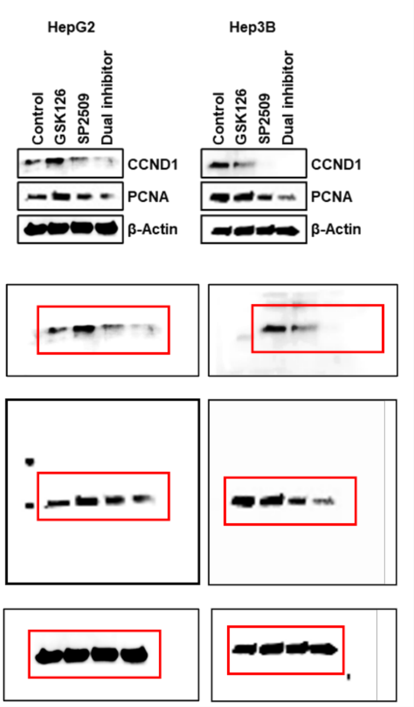

Figure 3E

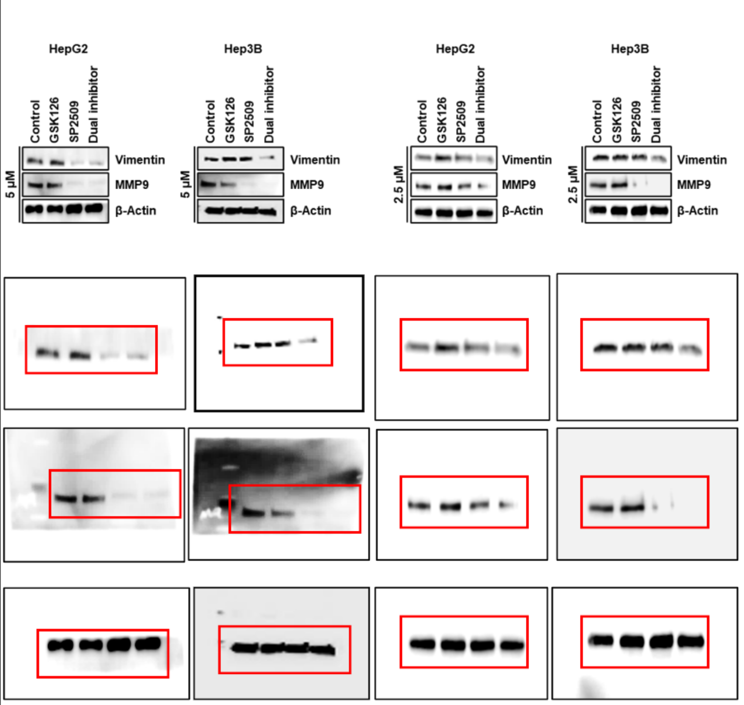

Figure 4B

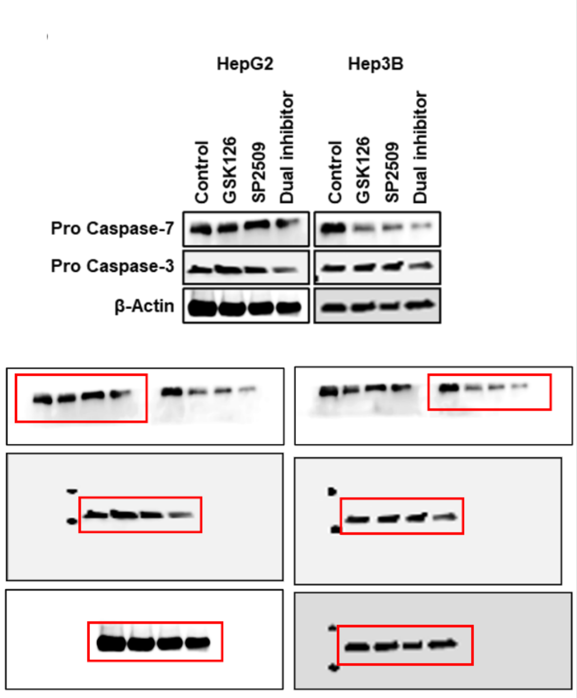

Figure 5C

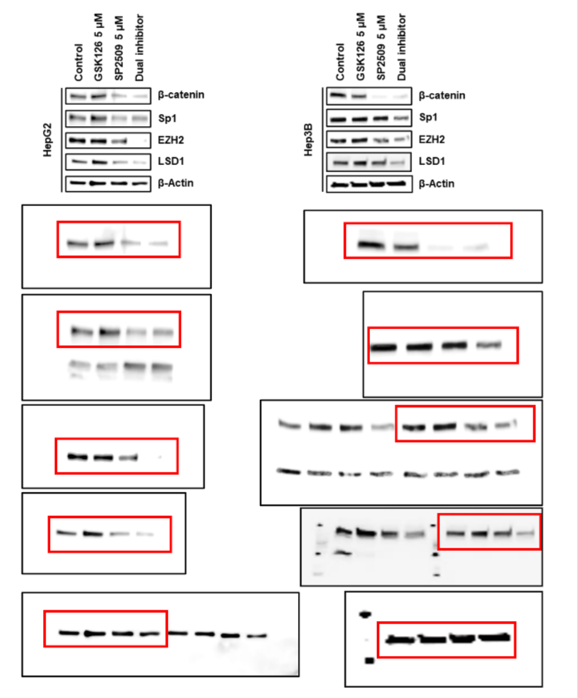

Supplementary Figure 14

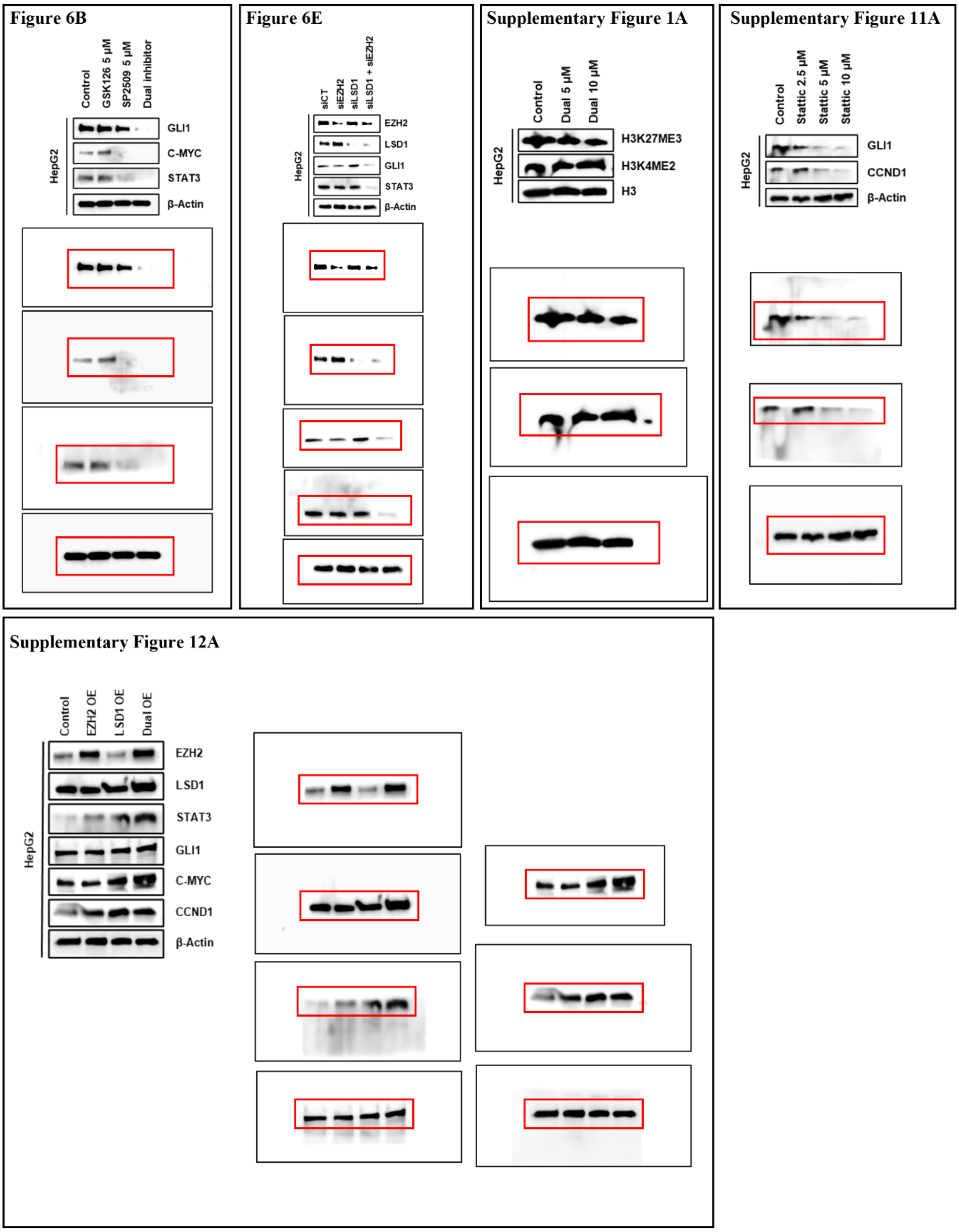

Supplement: Supplementary file 1 [file ijms-27-03886-s001.zip › ijms-3885813-supplementary.pdf]
